# Structure‐Activity Relationship for Di‐ up to Tetranuclear Macrocyclic Ruthenium Catalysts in Homogeneous Water Oxidation

**DOI:** 10.1002/chem.202100549

**Published:** 2021-05-27

**Authors:** Dorothee Schindler, Ana‐Lucia Meza‐Chincha, Maximilian Roth, Frank Würthner

**Affiliations:** ^1^ Institut für Organische Chemie Universität Würzburg Am Hubland 97074 Würzburg Germany; ^2^ Center for Nanosystems Chemistry (CNC) Universität Würzburg Theodor-Boveri-Weg 97074 Würzburg Germany

**Keywords:** homogeneous catalysis, metallomacrocycles, renewable fuels, ruthenium catalysts, water oxidation

## Abstract

Two di‐ and tetranuclear Ru(bda) (bda: 2,2′‐bipyridine‐6,6′‐dicarboxylate) macrocyclic complexes were synthesized and their catalytic activities in chemical and photochemical water oxidation investigated in a comparative manner to our previously reported trinuclear congener. Our studies have shown that the catalytic activities of this homologous series of multinuclear Ru(bda) macrocycles in homogeneous water oxidation are dependent on their size, exhibiting highest efficiencies for the largest tetranuclear catalyst. The turnover frequencies (TOFs) have increased from di‐ to tetranuclear macrocycles not only per catalyst molecule but more importantly also per Ru unit with TOF of 6 s^−1^ to 8.7 s^−1^ and 10.5 s^−1^ in chemical and 0.6 s^−1^ to 3.3 s^−1^ and 5.8 s^−1^ in photochemical water oxidation per Ru unit, respectively. Thus, for the first time, a clear structure–activity relationship could be established for this novel class of macrocyclic water oxidation catalysts.

## Graphical abstract

**A comparative study** on chemical and photochemical water oxidation with a homologous series of di‐, tri‐, and tetranuclear macrocyclic Ru(bda) complexes has shown that the catalytic efficiency of this novel class of water oxidation catalysts (WOCs) increases per Ru unit with the increasing macrocycle size, revealing the tetranuclear macrocycle as one of the best performing homogeneous WOCs.

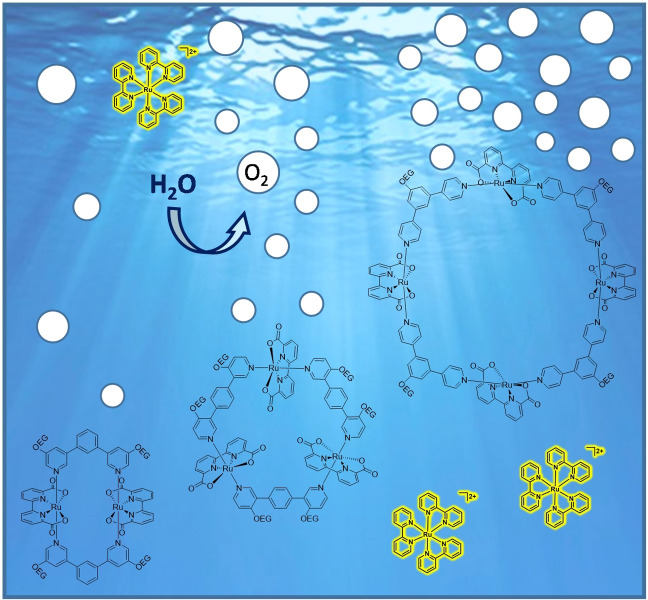

## Introduction

**Scheme 1:**
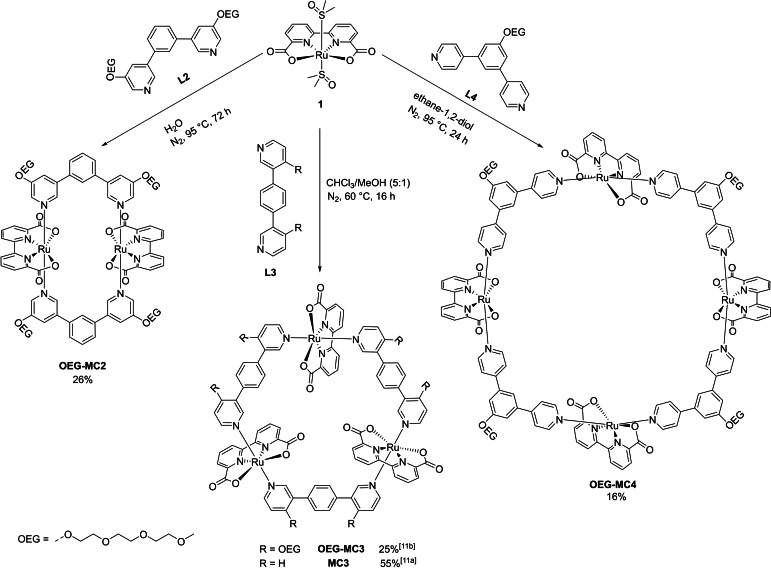
Synthesis of the new dimeric and tetrameric ruthenium macrocycles **OEG‐MC2** and **OEG‐MC4** as well as previously reported trimeric macrocycle **OEG‐MC3**.^[11b]^

One of the greatest challenges our society is facing today is achieving independence of finite fossil fuels and providing an environmental and climate benign energy supply.^[1]^ Consequences of burning oil, coal or natural gas such as harmful emissions and global warming have to be abated urgently to preserve our planet for future generations.^[2]^ In regard to an alternative energy supply, green hydrogen obtained by light‐driven splitting of abundant water is a promising solution.^[3]^ However, since the process of oxidizing water to provide the required protons and electrons for hydrogen formation is energetically a highly demanding process, catalysts are needed to overcome the overpotential of this reaction.^[4]^ Intensive research on water oxidation catalysts based on various transition metals has revealed that ruthenium catalysts are among the most efficient ones.^[5]^ Since the discovery of Ru(bda)(pic)_2_ (bda: 2,2′‐bipyridine‐6,6′‐dicarboxylate, pic: 4‐picoline) as a highly active water oxidation catalyst by Sun and co‐workers in 2009,^[6]^ several studies have been reported on diverse Ru(bda)‐based catalysts bearing modified axial and equatorial ligands.^[7]^ Dimeric,^[8]^ trimeric^[9]^ as well as polymeric^[10]^ structures have been obtained by assembly of multiple Ru(bda) units and axial ligands. In recent years, our group has developed a family of supramolecular macrocycles containing three Ru(bda) centers connected by 1,4‐bis(pyrid‐3‐yl) benzene (bpb) axial ligands and these trinuclear Ru macrocycles have been shown to be highly efficient water oxidation catalysts (WOCs).^[11]^ Notably, the reduced flexibility of the trinuclear macrocycles resulted in an enhanced stability as well as high turnover frequency (TOF) and turnover number (TON) values in chemical water oxidation, comparable to those of the oxygen evolving complex of photosystem II in nature.^[12]^ Detailed mechanistic studies confirmed a water nucleophilic attack (WNA) mechanism for the catalytic water oxidation with the unfunctionalized trinuclear macrocycle **MC3** (structure shown in Scheme 1)^[11a]^ in contrast to mononuclear complexes such as Ru(bda)(pic)_2_ that operate by binuclear I2M (interaction of two M−O units) mechanism.^[7a]^ The unequivocally confirmed WNA mechanism of macrocyclic Ru(bda) complexes makes them particularly interesting for applications under high dilution conditions and upon anchoring on surfaces.^[13]^ Based on theoretical calculations, a preorganized hydrogen‐bonded water network in the macrocyclic cavity of **MC3** has been proposed to explain its high catalytic activity in water oxidation.^[11c]^ Very recently, experimental evidence for the formation of such water networks has been obtained by X‐ray crystal structure analysis and X‐ray absorption studies performed on functionalized **MC3** derivatives.^[11e]^ Accordingly, efficient water oxidation by **MC3** and its derivatives is the result of cooperative proton abstraction during catalysis which leads to significant reduction of activation barriers for the key proton‐coupled electron transfer processes.[^11a^, ^11e^]

With Ru(bda) as a unique functional unit, we raised the question how the number of active centers within a multinuclear macrocycle would affect its catalytic efficiency. Although trinuclear macrocyclic Ru complexes have been reported for the first time in 2016,^[11a]^ and several noncyclic di‐ and trinuclear Ru WOCs have been developed in the past decade,[^8^, ^9^] reports on such cyclic Ru complexes are still scarce. Most recently, our group has reported a dinuclear macrocyclic Ru complex containing calix[4]arene‐based axial ligands that exhibits unexpectedly high catalytic performance in chemically‐driven and photocatalytic water oxidation.^[14]^ In the present study, hitherto unknown di‐ and tetranuclear Ru(bda) macrocycles bearing 1,3‐ and 1,4‐bipyridyl benzene axial ligands have been constructed by using non‐linear derivatives of the bridging ligands of **MC3** and the catalytic activities of the homologous series of di‐, tri‐, and tetranuclear macrocyclic complexes **OEG‐MC2**, **OEG‐MC3** and **OEG‐MC4** studied in a comparative manner. OEG (oligoethylene glycole) chains were introduced in macrocycles to enhance their solubility. Although the catalytic performance of **OEG‐MC3** has previously been studied in chemical water oxidation,^[11b]^ its photocatalytic activities remained unexplored and is accordingly also investigated in this work.

Our detailed studies have shown that the catalytic efficiency of the present series of macrocycles in chemical as well as photochemical water oxidation increases with the increasing size of the macrocycle with the highest TOF value of 42 s^−1^ in chemical and 23 s^−1^ in photochemical experiments for the tetranuclear WOC **OEG‐MC4**. Increasing catalytic efficiency has been observed not only for the molecular catalysts, but also per Ru unit of the multinuclear macrocycles. Thus, for the first time, a structure‐activity relationship has been established for this unique family of macrocyclic Ru(bda) WOCs.

## Results

### Synthesis of ligands and multinuclear cyclic Ru(bda) complexes

The to date unknown di‐ and tetranuclear Ru(bda) macrocyclic complexes **OEG‐MC2** and **OEG‐MC4** were synthesized using the axial bidentate ligands **L2** and **L4** having non‐linear arrangements of terminal pyridyl units with *meta* and *para* connectivity, respectively, at a benzene center (Scheme 1). The trinuclear macrocycle **OEG‐MC3** has been reported previously and was synthesized according to literature procedure.^[11b]^ As the solubility of metallomacrocycles decreases with increasing size,^[11b]^ solubilizing OEG chains were introduced to the axial ligands to facilitate the formation of the desired ruthenium macrocycles. The axial ligands **L2** and **L4** are literature‐unknown and were synthesized according to the routes displayed in the Supporting Information (Scheme S1).

The multinuclear Ru(bda) macrocycles were then synthesized by ligand exchange reactions of standard precursor complex Ru(bda)(dmso)_2_ **1** with the respective bidentate axial ligands (Scheme 1). Notably, under the previously established reaction conditions for the synthesis of **MC3** macrocycles[^11a^, ^11b^, ^11c^, ^11e^] including **OEG‐MC3**,^[11b]^ the reactions of **L2** and **L4** with the precursor complex **1** formed only mixtures of small oligomeric open chain products. Therefore, reaction conditions needed to be modulated to enable the formation of macrocyclic complexes using the nonlinear ligands **L2** and **L4**. Indeed, the reaction of Ru(bda)(dmso)_2_ **1** with pyridyl *meta*‐substituted axial ligand **L2** in a 1 : 1 ratio under nitrogen atmosphere in water at a higher temperature of 95 °C for 3 days afforded the dimeric Ru(bda) macrocycle **OEG‐MC2** (Scheme 1). Likewise, the assembly of *para*‐substituted **L4** and Ru(bda)(dmso)_2_ **1** under nitrogen atmosphere in ethylene glycol at 95 °C for 24 h led to the formation of the larger tetranuclear macrocycle **OEG‐MC4**. The separation of macrocyclic products from insoluble polymeric chains was achieved by column chromatography over Al_2_O_3_ and smaller oligomeric open chain side products could be removed by several size exclusion chromatography cycles over BioBeads SX1 or SX3 and subsequent gel permeation chromatography (GPC). Pure **OEG‐MC2** and **OEG‐MC4** were obtained in reasonably good isolated yields of 26 % and 16 %, respectively, for such macrocyclizations. Detailed synthetic procedures and characterization of the new ligands and macrocycles by NMR spectroscopy, mass spectrometry and CV analysis are reported in the Supporting Information (Figure S1–S12, S15–S27).

### Characterization of di‐ and tetranuclear macrocyclic Ru(bda) complexes

**Figure 1:**
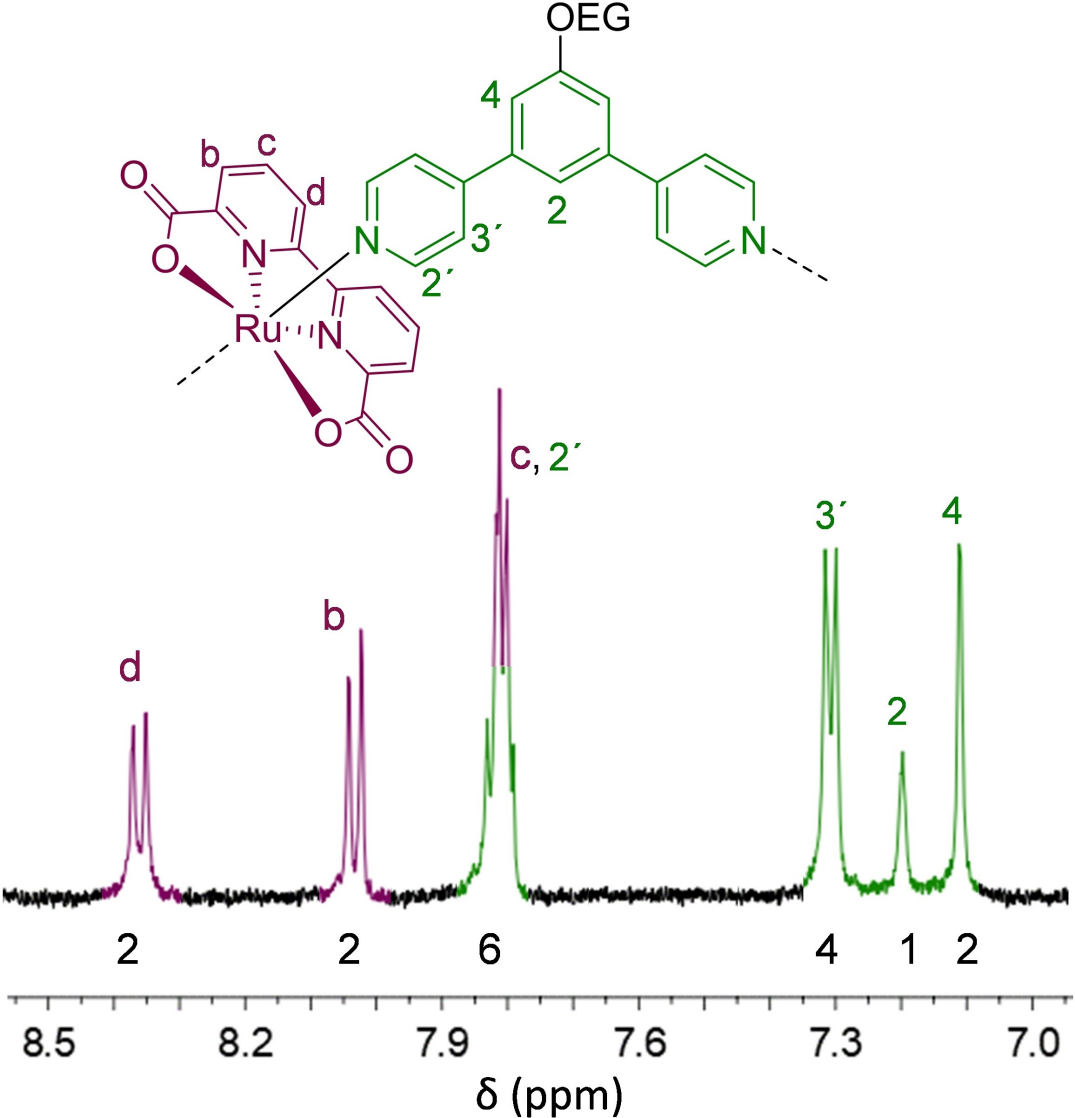
Aromatic region of ^1^H NMR spectrum of tetrameric **OEG‐MC4** in CD_2_Cl_2_/MeOD/CF_3_CD_2_OD 5 : 3 : 2 (400 MHz, rt), along with the structure of a monomeric repeat unit of this macrocycle. Numbers given under the signals correlate to the numbers of protons associated with each signal as determined by integration of the respective signal area. Colors of the signals correspond to bda (purple) and bpb (green) as highlighted in the structure.

The new Ru(bda) macrocycles were first characterized by ^1^H NMR spectroscopy. Traces of paramagnetic and NMR silent Ru^3+^ species were reduced to Ru^2+^ by addition of small amounts of ascorbic acid into the NMR tube to obtain high resolution spectra as exemplarily shown for **OEG‐MC4** (Figure 1 and Figure S7).

One single set of nicely resolved signals for the aromatic protons of the axial **L4** (shown in green) and equatorial bda ligands (shown in purple) of the tetrameric macrocycle **OEG‐MC4** are observed, thus excluding the presence of open chain side products. The fact that the pyridine protons of the axial ligands show only one set of signals clearly confirms the high symmetry and therefore macrocyclic structure of **OEG‐MC4**. In the case of linear oligomeric structures, multiple signals of these protons and additional signals of the non‐coordinated terminal pyridine rings or terminal Ru(bda) protons were to expect. The absence of such additional signals in the proton NMR spectra of **OEG‐MC4** as well as **OEG‐MC2** confirms the formation of macrocyclic structures (Figure S3 and S7). The smaller macrocycle **OEG‐MC2** could be further characterized by high‐resolution mass spectrometry (HR‐MS). A mass peak was observed for [M+Na]^+^ with *m/z*=1823.4219 (calculated 1823.4204), confirming the dimeric structure. Unfortunately, due to fragmentation of **OEG‐MC4** it was not possible to obtain a clean mass spectrum for the tetrameric macrocycle. However, upon deconvolution of signals the product peak could be detected as a weak signal but the intensity was not sufficient to enable reliable analysis of the isotope pattern.

**Figure 2:**
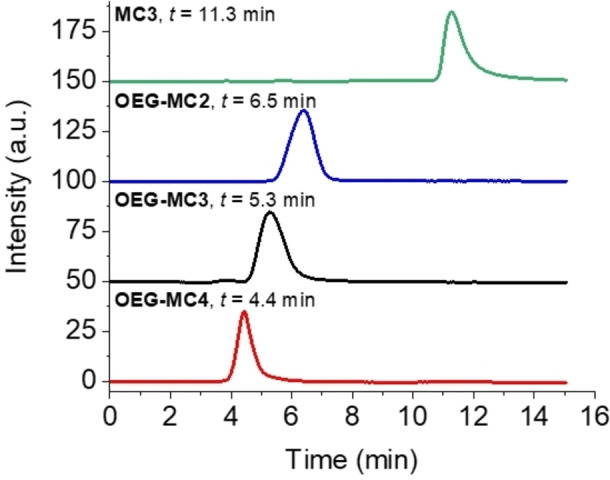
Analytical GPC chromatograms of **MC3** (green line), **OEG‐MC2** (blue line), **OEG‐MC3** (black line) and **OEG‐MC4** (red line).

Analytical GPC chromatograms of the purified products demonstrate the successful separation of macrocycles from smaller oligomeric side products as well as larger polymers and clearly indicate the different size of the new macrocycles compared to the literature‐known macrocycles **MC3** and **OEG‐MC3** (Figure 2).[^11a^, ^11b^, ^11c^] For GPC analysis, purified samples of di‐ and tetrameric OEG‐functionalized Ru(bda) complexes were dissolved in a CHCl_3_/MeOH (9 : 1) mixture and injected on an analytical GPC system with a SDV column (SDV: styrol‐divinylbenzol‐copolymer network). A comparison of the chromatograms of **MC3** and its OEG‐functionalized derivative **OEG‐MC3** reveals a significant influence of the OEG chains on the elution behavior of the macrocycle. The retention time of the **OEG‐MC3** with *t*=5.3 min is about half that of **MC3** without OEG functionalization (*t*=11.3 min). The shorter retention time (*t*=4.4 min) of the new tetrameric macrocycle **OEG‐MC4** than that of trimeric **OEG‐MC3** (*t*=5.3 min) indicates a slightly larger structure of the former, while the dimeric **OEG‐MC2** exhibits a longer retention time of *t*=6.5 min (Figure 2). The observed trend in retention times is in agreement with the expected increasing size from dinuclear to tetranuclear macrocycle as obtained by molecular modelling (MM2 optimization, Spartan’14, see Figure S13).

To further confirm the structures of the macrocycles, DOSY NMR spectroscopy of the series **OEG‐MC2**, **OEG‐MC3** and **OEG‐MC4** was performed (Figure S13). The acquired DOSY spectra show decreasing diffusion coefficients with the increasing size of the macrocycles from di‐, tri‐ to tetramer. Based on the diffusion coefficients, the hydrodynamic radius (${\;r}_{H}$ ) of each macrocycle was determined according to the Stokes‐Einstein equation (Eq. S1).^[15]^

Assuming a nearly spherical particle, the diffusion coefficient of *D*=‐10.09 m^2^ s^−1^ for **OEG‐MC4** results in a hydrodynamic radius of $r_{H}$ =2.7 nm (diameter *d_H_* =5.4 nm). Smaller hydrodynamic radii of 2.1 and 2.3 nm were obtained for **OEG‐MC2** and **OEG‐MC3**, respectively. The size of the macrocycles estimated from DOSY experiments corroborates very well with the trends obtained by molecular modelling (Figure S13), thus again confirming the macrocyclic structure of the multinuclear Ru(bda) complexes and their structural assignment.

### Optical and redox properties

**Figure 3:**
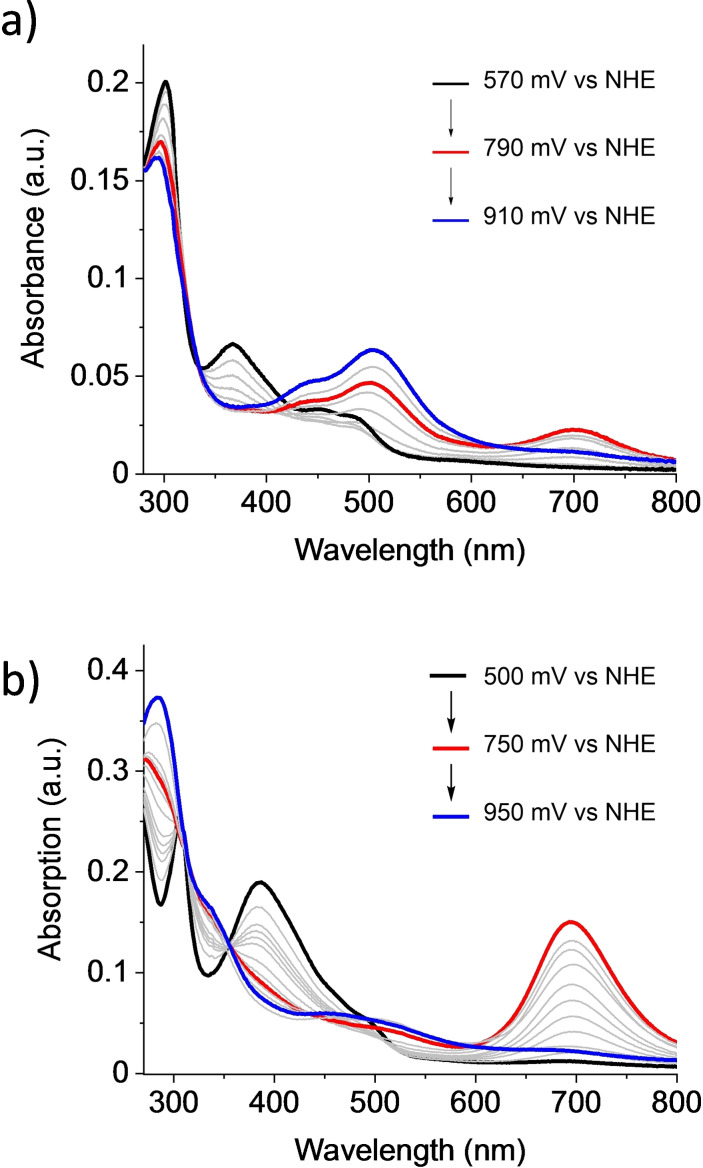
Spectroelectrochemical studies of a) **OEG‐MC2** (*c*=219 μM) and b) **OEG‐MC4** (*c*=250 μM) in a 1 : 1 mixture of H_2_O/TFE (phosphate buffer, pH 7, 0.1 M).

The optical properties of the new di‐ and tetrameric macrocycles **OEG‐MC2** and **OEG‐MC4** at different oxidation states were investigated by spectroelectrochemical measurements in phosphate buffer/trifluoroethanol (TFE) (1 : 1) at pH 7 (Figure 3). The spectroelectrochemical data for **OEG‐MC3** are shown in Figure S14d, exhibiting similar intensities and transitions as reported for trimeric macrocycle **MC3** without OEG.^[11e]^

The UV/vis absorption spectrum of **OEG‐MC2** at Ru^II^ state (Figure 3a, black line) shows a relatively intense sharp band at 300 nm, which is typical for Ru complexes and attributed to the axial ligand‐centered π‐π* transition.^[7c]^ The two broad bands at lower energies at about 370 nm and 450 nm correspond to metal‐to‐ligand charge transfer (MLCT) transitions from the Ru‐d orbital to the π* orbitals of the axial and bda ligand.[^7c^, ^16^] Calculations have shown that the Ru‐d to bda‐π* MLCT bands appear at lower energies compared to Ru‐d to L‐π* (L=axial ligand).^[17]^ Similar transitions were observed for **OEG‐MC4**, where the axial ligand‐centered π‐π* transition appears at about 310 nm (Figure 3b). In this case, Ru‐d to L‐π* transitions are not distinguishable from Ru‐d to bda‐π* due to overlapping of the signals and thus detected as one broad band from 340 nm to 530 nm with a maximum intensity at 385 nm. It is known that each Ru oxidation state has a distinctive UV/vis absorption spectrum.^[11c]^ By applying an increasing voltage, stepwise oxidation of the Ru centers from Ru^II^ to Ru^IV^ could be observed in these complexes. Changes in the UV/vis spectra were observed during the transformation to the Ru^III^ state by a significant decrease in intensity of the MLCT transition between 340 nm and 430 nm of both macrocycles. Additionally, a new band arises with a maximum at 700 nm which is of lower intensity in the case of the dinuclear macrocycle and much more distinct for the tetranuclear one (Figure 3b). This absorption band was also observed for the trinuclear non‐functionalized macrocycle **MC3**. Earlier studies of a series of homologous trinuclear Ru(bda) macrocycles with different sized cavities indicate a correlation between the intensity of this band and the activity of the respective catalysts.^[11c]^ Detailed theoretical calculations revealed that this band does not originate from Ru^III^−O−Ru^III^ complexes but can be assigned to the Ru(bda)‐σ to Ru(bda)‐σ* transition in the Ru^III^ state.^[11d]^ In addition, it has been reported that the strong absorption at 700 nm depends on the distance of a coordinated aqua / hydroxyl molecule to the Ru center.^[11c]^ It is assumed that, due to an equilibrium between [Ru^III^]^+^ and [Ru^III^‐OH_2_]^+^ species,^[18]^ the existence of this band and its intensity depends on whether the six or seven coordinated form is favored. Simulations for the monomeric Ru(bda)(pic)_2_ complex demonstrated that no transition appears at 700 nm as the arrangement of the axial ligands is blocking the 7^th^ coordination site at the Ru center and making it difficult for a water molecule to access the metal center. In contrast to monomeric Ru(bda)(pic)_2_, macrocycle **MC3** has a relatively fixed axial ligand which facilitates the coordination of water leading to an intense absorption band at 700 nm as reported previously.^[11c]^ The OEG‐functionalized derivative **OEG‐MC3** shows similar spectroelectrochemical behavior (Figure S14d) as parent **MC3**. For all these macrocycles, the band at around 700 nm decreases upon further oxidation and finally vanishes completely when reaching the Ru^IV^ state at higher potentials (from 910 to 1020 mV vs. NHE, see Figure 3 and S14d).

The redox properties of the macrocycles were studied by cyclic voltammetry (CV) and differential pulse voltammetry (DPV) in a 1 : 1 phosphate buffer/TFE mixture at pH 7 (Figure S15–S27). The addition of the non‐coordinating co‐solvent TFE was needed to circumvent the poor solubility in pure water and is reported to have no significant effect on the redox potentials.^[11c]^ For **OEG‐MC2**, three redox events were observed at +0.66 V, +0.82 V and +1.07 V that can be assigned to the Ru^III^_2_/Ru^II^_2_, Ru^IV^_2_/Ru^III^_2_, and Ru^V^_2_/Ru^IV^_2_ oxidation processes, respectively. For comparison, **OEG‐MC4** showed redox processes at +0.67 V, +0.84 V and +1.03 V. A comparison of the redox potentials of di‐ and tetranuclear macrocycles with those of trinuclear macrocycles with and without OEG chains revealed nearly identical oxidation potentials of all macrocycles, indicating that the different spatial arrangement of the Ru(bda) units in a di‐, tri‐ or tetranuclear complex as well as the introduction of OEG chains have only a minor effect on the redox properties of the Ru center (Table 1). Importantly, since the Ru^V^/Ru^IV^ oxidation potential of the macrocycles appears below the Ru^III^/Ru^II^ oxidation potential of Ru(bpy)_3_^2+^ (*E*=+1.26 V vs. NHE),^[19]^ they appear properly suited for photocatalytic water oxidation driven by Ru(bpy)_3_^2+^ as a photosensitizer.

**Table 1:** Comparison of redox properties of macrocyclic Ru(bda) complexes **MC3**, **OEG‐MC2**, **OEG‐MC3** and **OEG‐MC4** under neutral conditions.^[a]^

WOC	*E* vs. NHE (V)		
	Ru^III/II^	Ru^IV/III^	Ru^V/IV^
**MC3**[43]	+0.66	+0.82	+1.00
**OEG‐MC2**	+0.66	+0.82	+1.07
**OEG‐MC3**	+0.67	+0.81	+1.01
**OEG‐MC4**	+0.67	+0.84	+1.03

[a] Data extracted from DPV experiments in 1 : 1 mixture of H_2_O/TFE (phosphate buffer, pH 7, 0.1 M), *c*(WOC)=0.25 mM.

### Chemical water oxidation

The catalytic performance of new di‐ and tetranuclear Ru(bda) macrocycles was first studied in chemical water oxidation using ceric ammonium nitrate (CAN) as a sacrificial electron acceptor. Although the use of a sacrificial oxidant to drive the water oxidation reaction does not mimic conditions that are required for artificial photosynthesis, it allows for an initial comparison of trends in catalytic activities of the WOCs. In general, water oxidation is known to be favored at higher pH which enables reduction of overpotentials.^[7d]^ However, chemical water oxidation is commonly performed applying the strong oxidant CAN which is only stable in acidic media, particularly at pH 1.^[20]^ Water oxidation experiments with the OEG‐WOCs presented in this work were performed in air‐tight Schlenk flasks attached to pressure sensors and equipped with a septum for injection of the catalyst. A fresh solution of excess CAN was prepared in acidic aqueous mixtures (triflic acid) containing 50 % acetonitrile as a co‐solvent, which was reported to be robust in highly oxidizing environments.^[11c]^

**Figure 4:**
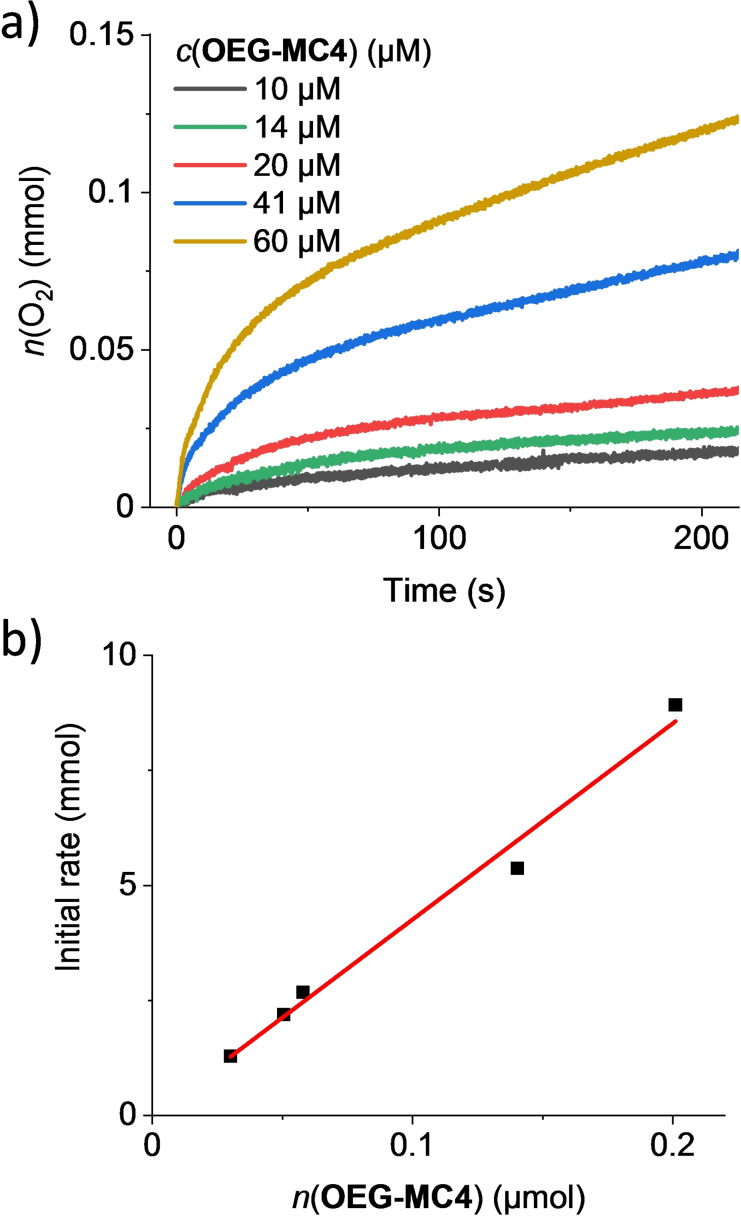
a) Concentration‐dependent water oxidation experiments with **OEG‐MC4** as WOC in H_2_O/MeCN 1 : 1 (pH 1, triflic acid). b) Plot of initial rates vs. catalyst amount with linear regression for the determination of TOF.

Oxygen evolution curves at different concentrations for **OEG‐MC4** are depicted exemplarily in Figure 4a (see Figure S28 for **OEG‐MC2**). For both new catalysts, an increase in pressure was observed immediately after addition of CAN. Initial rates of the water oxidation were determined in the first two seconds of catalysis and a linear dependency of the rate on the concentration of the catalyst was observed (Figure 4b). This first order kinetics is typically found for catalysts operating via WNA mechanism as previously demonstrated for trinuclear WOC **MC3**.^[11a]^ In contrast, for catalysts working via I2M mechanism, for example monomer Ru(bda)(pic)_2_, rates increase faster and in a non‐linear fashion for higher catalyst concentrations.[^11a^, ^21^]

Average TOF values per macrocyclic catalyst were calculated from the slope of the linear regression of the initial rates vs. catalyst concentration. A TON was determined at each concentration and the reported value represents the maximal TON (highest TON observed at one concentration during concentration‐dependent measurements) obtained for each catalyst. For tetranuclear **OEG‐MC4**, an average TOF of (42±3) s^−1^ was obtained, which is considerably higher than the previously reported TOF of 26 s^−1^ for **OEG‐MC3**,^[11b]^ while dinuclear **OEG‐MC2** showed a significantly lower activity than the tetranuclear WOC with TOF=(12±2) s^−1^. Thus, a clear trend in increasing catalytic activities with increased size of the macrocycles is evident not only per macrocycle but also per Ru unit with TOFs per Ru of 6 s^−1^, 8.7 s^−1^ and 10.5 s^−1^ for the di‐, tri‐ and tetranuclear macrocycles, respectively. However, the stability that is reflected in TON values, is similar for **OEG‐MC3** and **OEG‐MC4** (2200 and 2870, respectively), whereas **OEG‐MC2** exhibits a much lower value of only 200. Analysis of the headspace of the reaction vessel by gas chromatography was performed to determine the gas composition after each experiment (Figure S29 and S30). For both macrocycles **OEG‐MC2** and **OEG‐MC4**, the amount of oxygen detected by gas chromatography is in agreement with the amount calculated from the pressure sensor detection. These findings demonstrate that under the conditions applied in catalytic water oxidation with these WOCs only oxygen and no other gaseous by‐products are generated.

### Photocatalytic water oxidation

**Figure 5:**
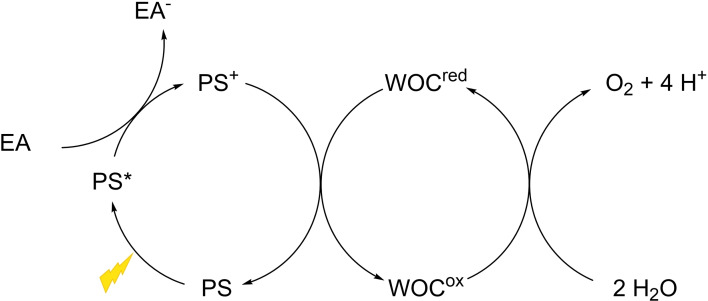
Schematic illustration of photocatalytic water oxidation using a one‐electron transferring PS, a sacrificial EA and a WOC.

One step closer to mimicking natural photosynthesis in an artificial approach is the investigation of WOCs in photocatalytic water oxidation.^[22]^ A typical light‐driven experiment for studying the catalytic activities of WOCs requires the use of a three component system consisting of the WOC, a photosensitizer (PS) and a sacrificial electron acceptor (EA) (Figure 5). The process starts with the absorption of solar irradiation by the PS. As the PS transfers an electron to an EA, it increases its oxidizing power and is able to activate the catalyst.

The catalytic activities of the OEG‐functionalized macrocycles **OEG‐MC2**, **OEG‐MC3** and **OEG‐MC4** were investigated using Ru(bpy)_3_^2+^ as PS and Na_2_S_2_O_8_ as EA under neutral conditions (pH 7).^[23]^ As mentioned before, chemically driven catalytic water oxidation of **OEG‐MC3** has been previously investigated,^[11b]^ but its photocatalytic activities remained unexplored. Thus, this macrocycle is also included in our studies. To generate the mild oxidant Ru(bpy)_3_^3+^ (PS^+^) in situ from Ru(bpy)_3_^2+^, the PS is exposed to light, leading to the formation of the excited ^3^MLCT state PS*.^[24]^ By one‐electron transfer to the EA, PS^+^ is generated and Na_2_S_2_O_8_ is converted into SO_4_^2−^ anion and SO_4_^•−^ radical. The latter can react with another PS molecule in the ground state and generate an additional PS^+^. With the Ru^III/II^ redox potential of the PS at +1.26 V vs. NHE,^[19]^ the oxidative power of PS^+^ is sufficient to enable consecutive oxidation of the WOCs to the Ru^V^ state where water oxidation is initiated and the PS is regenerated.

**Figure 6:**
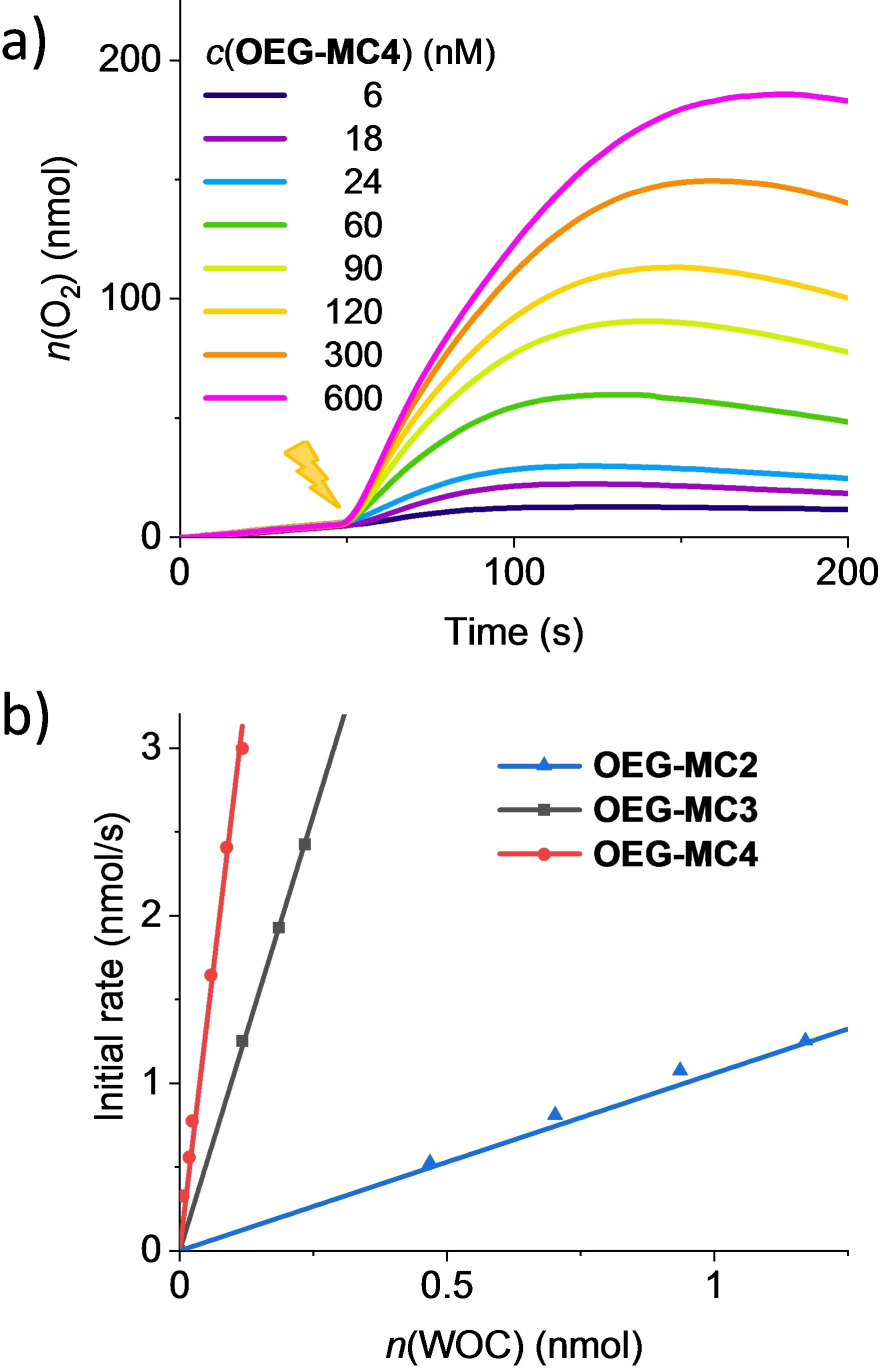
a) Oxygen evolution curves of **OEG‐MC4** at different concentrations in H_2_O/MeCN 1 : 1 (pH 7, phosphate buffer), *c*(PS)=1.5 mM, *c*(Na_2_S_2_O_8_)=37 mM. The lighting symbol indicates the start of sample irradiation at *t*=50 s. b) Plots of initial rates vs. concentrations of macrocycles studied here with linear regression for the determination of averaged TOF.

Light‐driven water oxidation experiments were performed at 20 °C in a temperature‐controlled transparent reaction chamber, which was connected to a Clark electrode set‐up for the detection of generated oxygen. For irradiation of the samples, a xenon lamp with calibrated intensity of sunlight at the earth surface (100 mW/cm^2^)^[25]^ was employed. To overcome the poor solubility of the WOCs in pure water, acetonitrile was chosen as co‐solvent due to its stability in oxidative environments.^[11c]^ Experiments were performed at pH 7 in a phosphate buffer/MeCN mixture (1 : 1) under identical conditions to ensure reliable comparability.^[11e]^ Concentrations of PS (1.5 mM) and sodium persulfate as EA (37 mM) were kept constant in all experiments, while the concentration of the WOC was varied. In a standardized procedure, PS and EA were mixed with the particular amount of WOC in the dark and irradiation was started after 50 s. When the solution was mixed in the dark, no oxygen was generated. However, after illumination of the sample and a short induction period of about 1–2 s, an increase in oxygen concentration was clearly detected by the Clark electrode. Oxygen evolution curves at different concentrations of the WOC, as shown for **OEG‐MC4** in Figure 6a, were observed for all the investigated macrocycles. Experiments for **OEG‐MC2** and **OEG‐MC3** are shown in Figure S31 and S32.

After the rate of oxygen production subsides, a plateau is observed as no further oxygen is generated. A slight decrease in the oxygen evolution curves is then observed as some of the dissolved oxygen is released into the gas phase or some oxygen is reduced by the photosensitizer after deactivation of the catalyst. By increasing the concentration of the catalyst, a higher amount of photo‐generated O_2_ is detected and steeper curves are observed (Figure 6a). Initial rates for each measurement at different concentrations were determined from the linear increase of the oxygen evolution curves in the first 10 to 15 s of catalysis (for further details see “Materials and methods” in the Supporting Information). A linear dependency of the rates on the amount of WOC was observed for all the macrocycles (Figure 6b). By linear regression in the plot of initial rates at different concentrations an average TOF value was obtained for each catalyst. TON values were calculated from the maximum amount of oxygen detected at each concentration. The reported TON reflects the highest value obtained for each catalyst. Table 2 summarizes the photocatalytic activities of the OEG‐functionalized macrocyclic Ru(bda) WOCs in comparison to the unfunctionalized macrocycle **MC3** ^[11e]^ previously investigated by our group under identical conditions.

**Table 2:** Catalytic data of macrocyclic WOCs in photocatalytic water oxidation.^[a]^

WOC	TOF [s^−1^]	TOF [s^−1^]/Ru	TON
**OEG‐MC2**	(1.1±0.1)	0.6	36
**OEG‐MC3**	(10±0.4)	3.3	400
**OEG‐MC4**	(23±0.7)	5.8	500
**MC3**[43]	(11±0.5)	3.7	430

[a] Photochemical water oxidation in H_2_O : MeCN 1 : 1 (pH 7, phosphate buffer), *c*(PS)=1.5 mM, *c*(Na_2_S_2_O_8_)=37 mM, *c*(WOC)=6 nM–6 μM.

Tetrameric macrocycle **OEG‐MC4** is the most active catalyst among the present series as evident from its steeper linear regression in Figure 6b. Oxygen evolution was already detected at remarkably low catalyst concentration of 6 nM. With a TOF of 23 s^−1^, this tetranuclear complex is more than twice as active as trimeric **OEG‐MC3** with a TOF of 10 s^−1^, whereas dimeric **OEG‐MC2** shows with TOF=1.1 s^−1^ one order of magnitude lower activity than the larger macrocycles. TOFs of these macrocycles per Ru unit (see Table 2) reflect the same trend of increasing activity in the larger macrocycles. Additionally, a TON of 36 indicates the lower stability of **OEG‐MC2** compared to **OEG‐MC3** and **OEG‐MC4** (TON=400 and 500, respectively), which are in a similar range as the unfunctionalized macrocycle **MC3**.^[11e]^ As observed for chemical oxidation, the efficiencies of OEG‐functionalized macrocyclic WOCs in photocatalytic water oxidation increased with increasing size not only per catalyst molecule but also per Ru unit and thus a clear structure‐activity relationship is given.

## Discussion

Following the successful synthesis of di‐ and tetranuclear Ru(bda) macrocycles **OEG‐MC2** and **OEG‐MC4**, the catalytic performance of these WOCs, along with our previously reported trinuclear **OEG‐MC3**,^[11b]^ both in chemical and light‐driven water oxidation has been explored in a comparative manner. Albeit water oxidation starts at higher voltages at acidic pH, chemically induced water oxidation at pH 1 is a useful tool for gaining first information on activities of catalysts.^[20]^ However, it should be noted that WOCs display different activities under various conditions and low activities in chemically driven water oxidation might not rule out high performances in photocatalysis or electrocatalysis. New macrocycles **OEG‐MC2** and **OEG‐MC4** show TOFs of 12 s^−1^ and 42 s^−1^ in chemical oxidation at pH 1, which meets the expectation of higher activities in catalysts with more active centers. Interestingly, TOFs per Ru(bda) unit also reveal the same trend as TOF values of 6 s^−1^, 8.7 s^−1^ and 10.5 s^−1^ were obtained for the di‐, tri‐ and tetranuclear OEG‐WOCs, respectively. TONs, which represent stability, are not increased with the size of the macrocycle as similar TON values were observed for the tri‐ and tetranuclear WOCs **OEG‐MC3** and **OEG‐MC4** (TON=2200 and 2870), while dinuclear **OEG‐MC2** showed much lower TON of 200.

Similar trends were observed in photocatalytic water oxidation at neutral pH. The catalytic activity of **OEG‐MC4** with a TOF of 23 s^−1^ is among the highest reported for a Ru(bda) complex in photocatalysis, being only outperformed by a Ru^IV^=O(tda) complex, which reaches a TOF of 50 s^−1^.^[26]^ To put this into context, the values should be compared to those of Ru(bda)(pic)_2_ and the unsubstituted **MC3**.^[11e]^ For the latter, a TOF of 11 s^−1^ in photocatalytic water oxidation was reported which is comparable to the activity of the trinuclear macrocycle **OEG‐MC3** with the identical basic framework exhibiting a TOF of 10 s^‐1^. For the monomeric Ru(bda)(pic)_2_, a TOF of 0.6 s^−1^ was observed (Figure S33). Considering the presence of two Ru(bda) active centers in **OEG‐MC2**, the catalytic activity of this dinuclear complex with TOF of 1.1 s^−1^ (0.6 s^−1^ per Ru unit) is nearly identical with that of monomeric Ru(bda)(pic)_2_. Therefore, no gain in catalytic performance could be achieved by macrocyclic arrangement in dimer. Thus, it can be assumed that each catalytically active Ru center in **OEG‐MC2** functions independently. On the other hand, the TOFs per Ru unit for **OEG‐MC4** (5.8 s^−1^) and **OEG‐MC3** (3.3 s^−1^) are significantly higher than that of monomeric Ru(bda)(pic)_2_ and **OEG‐MC2**, indicating beneficial preorganization of catalytically active Ru(bda) units in larger macrocycles leading to higher catalytic activities.

Interestingly, **OEG‐MC2** shows a higher TON per Ru unit (TON=18) than Ru(bda)(pic)_2_ (TON=13) indicating that the dimeric macrocycle is more stable than the monomer. This can presumably be attributed to the rigid nature of the bridging ligand. Due to the chelating effect of **L2**, axial pyridyl ligand dissociation, which is known to be one of the main degradation pathways for molecular Ru(bda) WOCs,^[27]^ can be diminished as self‐healing processes take place by a re‐association. Both tri‐ and tetranuclear complexes **OEG‐MC3** and **OEG‐MC4** feature similar stabilities as **MC3** with TONs of 133, 125 and 143 per Ru center, respectively, that are remarkably higher than those of dinuclear **OEG‐MC2** and monomeric reference catalyst Ru(bda)(pic)_2_. These results imply a significant gain in stability by the macrocyclic effect compared to the monomer.

These results are supported by spectroelectrochemical investigations that revealed intense signals for the Ru^III^ state at 700 nm, which are associated with the influence of a hydrogen‐bonded water network within the cavity as reported previously for **MC3**.^[11c]^ Very recently, we reported of experimental evidence of this water network in substituted **MC3** macrocycles. Electron density in X‐ray diffraction of single crystaly could be assigned to water molecules and was found to be in very good agreement with theoretical studies and molecular dynamic simulations.[^11c^, ^11e^] The fact that only a very weak band was observed in the case of **OEG‐MC2** corroborates our hypothesis that the intensity of this band relates to the catalytic activity for water oxidation. Thus, the increasing intensity of the 700 nm band for the Ru^III^ state correlates with the increasing catalytic activity from di‐ to tetranuclear complex with TOFs per Ru center of 6 s^−1^, 8.7 s^−1^ and 10.5 s^−1^ using CAN as an oxidant at pH 1 in 50 % water/MeCN. TON values show a similar trend with the highest TON of 2870 observed for **OEG‐MC4**, but comparison with the unsubstituted **MC3** ^[11e]^ indicates that the harsh conditions in acidic solution are disadvantageous for the OEG‐substituted WOCs and presumably lead to oxidative degradation.

## Conclusions

Mononuclear water oxidation catalysts containing Ru(bda) as an active unit have been studied extensively during the last decade. However, not many reports on multinuclear Ru(bda) WOCs are known. In this work, new dinuclear and tetranuclear macrocyclic WOCs **OEG‐MC2** and **OEG‐MC4** were synthesized with implemented Ru(bda) units and triethylene glycol chains in axial ligands to enhance solubility of the metallosupramolecular structures in aqueous media. ^1^H NMR spectra of **OEG‐MC2** and **OEG‐MC4** revealed a highly symmetric structure of these compounds, demonstrating the absence of open chain ends. The different size of the macrocycles compared with the previously reported **OEG‐MC3** was confirmed by analytical GPC and DOSY NMR spectroscopy. Redox properties and catalytic activities of the macrocyclic WOCs in chemically and light‐driven water oxidation have been investigated. While the redox potentials of these macrocycles for the oxidation of the Ru centers from Ru^II^ to Ru^IV^ are rather similar, differences in spectroelectrochemical features could be observed, in particular a characteristic band at 700 nm whose intensity correlates with the catalytic activity for these macrocycles. In light‐driven catalytic water oxidation with the present series of macrocyclic ruthenium WOCs under neutral conditions (pH 7) similar trends were observed as for chemical water oxidation. **OEG‐MC2** performed with similar efficiency per Ru unit as the monomeric reference catalyst Ru(bda)(pic)_2_. Pleasingly, much better performances were observed for tri‐ and tetranuclear OEG‐WOCs. **OEG‐MC4** with a TOF of 23 s^−1^ (5.8 s^−1^ per Ru unit) belongs to the best performing homogeneous Ru(bda) WOCs in photocatalytic water oxidation and thus possesses potential for application in artificial photosynthesis devices.

## Conflict of interest

The authors declare no conflict of interest.

## Supporting information

As a service to our authors and readers, this journal provides supporting information supplied by the authors. Such materials are peer reviewed and may be re‐organized for online delivery, but are not copy‐edited or typeset. Technical support issues arising from supporting information (other than missing files) should be addressed to the authors.

Supporting Information
