# Supplementary material for: Structure‐Activity Relationship for Di‐ up to Tetranuclear Macrocyclic Ruthenium Catalysts in Homogeneous Water Oxidation
Source: Chemistry. 2021 May 27;27(68):16938–46. doi: 10.1002/chem.202100549 (PMC9290496; doi:10.1002/chem.202100549)
Supplement: Supplementary file 1 — Supporting Information [file CHEM-27-16938-s001.pdf]

Chemistry–A European Journal

Supporting Information

Structure-Activity Relationship for Di- up to Tetranuclear Macrocyclic Ruthenium Catalysts in Homogeneous Water Oxidation

Dorothee Schindler, Ana-Lucia Meza-Chincha, Maximilian Roth, and Frank Würthner*

Supporting Information

Table of Contents

Materials and methods 2

Synthesis of ligands and macrocyclic WOCs 5

NMR spectra 10

DOSY NMR spectra 15

UV/vis absorption spectroscopy and spectroelectrochemical measurements 16

Electrochemical studies 16

Chemical water oxidation 21

Photocatalytic water oxidation 22

References 23

Materials and methods

All chemicals and reagents were purchased from commercial suppliers and used without any further purification, unless noted otherwise. Compounds 2,2'-bipyridine-6,6'-dicarboxylic acid (bda),[1] $RuCl_2(dmso)_4$,[2] Ru(bda)$(dmso)_2$,[3] Ru(bda)$(pic)_2$,[4] **L3**,[5] **OEG-MC3**[6] and **MC3**[9] were synthesized according to previously published procedures. $[Ru(bpy)_3]Cl_2$ was purchased from Sigma Aldrich with 99.95% purity. Deuterated solvents for NMR analysis were purchased from Deutero (MeOD, CD_2Cl_2) or Eurisotop (TFE-d_3). Unless otherwise noted, reactions were carried out under normal laboratory atmosphere. Reactions with oxygen or water sensitive reagents were carried out under a nitrogen atmosphere. For homogeneous CV as well as water oxidation experiments and reactions in aqueous media highly pure water obtained from a PURELAB Classic water purification system (ELGA) was used. Phosphate buffer for light-induced water oxidation experiments and UV/Vis studies at pH 7 was bought from Honeywell. All co-solvents used in electrochemical studies were of a purity of 99.8% and higher.

Size exclusion chromatography (GPC)

Size exclusion chromatography was performed in a 9:1 DMC/MeOH mixture on suspended BioBeads particles (S-X1, Bio-Rad). Analytical GPC was performed on a JASCO system (RI-2031 Plus Intelligent RI Detector MD-2015 Plus Multiple wavelength detector, LG-2080-02 Ternary Gradient Unit and one PSS SDV 5µ 8x30 mm linear M column). Preparative GPC purification was performed on a LC-5060 system of Japan Analytical Industries (three columns from Agilent, PLgel, 10 µm, 100 Å; 10 µm, 100 Å; 10 µm, 500 Å). In both cases, a mixture of chloroform (90%) stabilized with ethanol and methanol (10%) was used as eluent.

NMR spectroscopy

^{1}H NMR spectra were recorded at room temperature (ca. 22 °C) with a Bruker Avance III HD 400 spectrometer at 400 MHz. ^{13}C NMR spectra are proton decoupled and recorded at 100 MHz using the same instrument. DOSY spectra were recorded on a Bruker Avance III HD 600 spectrometer. Chemical shifts δ are indicated in parts per million (ppm) relative to the solvent peaks and coupling constants J in Hz. Solvent peaks of residual undeuterated solvent were used for calibration of the spectra. The following abbreviations were used to describe the observed multiplicities: s = singlet, d = doublet, t = triplet, m = multiplet, dd = doublet of a doublet.

DOSY NMR

DOSY (diffusion ordered spectroscopy) spectra were recorded at 295.2 K in a mixture of deuterated 2,2,2-triflouroethanol/dichloromethane/methanol (10:3:7). The viscosity of η = 0.99 $*10^{-3}\,\frac{\mathrm{kg}}{\mathrm{m\,s}}$ of the used solvent mixture was determined using a Ubbelohde Viscometer (type 501

03). The hydrodynamic radius of the sample assuming a spherical character was estimated using the Stokes-Einstein equation (equation 1).

$$D = \frac{k\,T}{6\,\pi\,\eta\,r_H} \qquad \text{(eq. S1)}$$

Here, D is the diffusion coefficient which was determined from the DOSY experiments, k is the Boltzman constant, T is the temperature and η is the dynamic viscosity.

Mass spectrometry

High-resolution mass spectra (HR-ESI) were recorded on an ESI microTOF Focus mass spectrometer from Bruker Daltonics GmbH. Measurements were carried out in positive mode and mass signals are reported as fragment mass per charge (*m*/*z*).

Melting points

Melting points were measured in open glass capillaries in a melting point apparatus (Büchi) or a BX41 optical microscope (Olympus) and are not corrected.

UV/vis absorption spectroscopy

UV/vis absorption spectra were recorded in 1 cm quartz cuvettes with a Jasco V-670 spectrometer at 25 °C using spectroscopic grade solvents.

Electrochemical measurements

Cyclic voltammetry (CV) or differential pulse voltammetry (DPV) experiments were carried out on a BAS Epsilon (BAS Cellstand C3) potentiostat. A standard three electron configuration was used with a glassy carbon disk (ϕ = 0.3 cm, S = 0.07 cm^2) as working electrode (WE), platinum wire (homogeneous measurements) as a counter electrode (CE) and Ag/AgCl (3 M KCl) as a reference electrode (RE). Samples were measured in homogeneous phase and prepared in 1:1 water/TFE mixtures (pH 7 phosphate buffer, 0.1 M). All potentials were converted and are reported vs normal hydrogen electrode (NHE). For ensuring the same distance of the electrodes in CV and DPV measurements, a glass vial with a self-made Teflon lid with three holes for the electrodes was used. CV experiments were recorded at a scan rate of 100 mV/s with an *iR* compensation of 85%. The following parameters were used in DPV experiments: ΔE = 4 mV, amplitude = 50 mV, pulse width = 0.05 s, sampling width = 0.0167 s and pulse period = 0.2 s.

Spectroelectrochemistry

Measurements were performed in reflection mode using Agilent Cary 5000 spectrometer. A home built cylindrical PTFF cell with a sapphire window and an adjustable three-in-one electrode setup comprising a 6 mm platinum disk as a working electrode, a 1 mm platinum counter electrode and a pseudo-reference electrode was used. The layer thickness was set to be about 100 µm and experiments were performed in 1:1 TFE/water mixtures (pH 7, phosphate buffer). Potentials were referenced to the first oxidation event determined by DPV.

Chemical water oxidation

For chemically driven water oxidation experiments, gas tight reaction vessels of V = 20.6 mL were connected to pressure transducers (Honeywell, SSCDANN030PAAA5, absolute pressure, 0 to 30 psi). In one single experiment, cer ammonium nitrate (CAN, 1 g, 1.82 mmol) was dissolved in 3 mL of a water/acetonitrile mixture (pH 1, triflic acid, various ratios) in the reaction vessel and 400 µL of a stock solution of the catalyst dissolved in water/acetonitrile (pH 1, triflic acid, same ratio as in experiment) were injected through a septum using a Hamilton syringe. At the end of each experiment, 500 µL gas of the head space was taken with a gas tight Hamilton syringe and injected into a gas chromatograph GC-2010 Plus (Shimadzu, thermal conductivity detector at 30 mA, argon as carrier gas) for analyzing its composition. For calculating the TON, the total amount of generated oxygen during catalysis was determined and divided by the amount of catalyst present in the experiment. By applying the ideal gas law, the quantity of evolved oxygen was determined from the increase in pressure in the reaction vessel:

$$\Delta p * V = R * T * \Delta n \qquad \text{(eq. S2)}$$

where Δp is the pressure increase, V is the volume of the reaction vessels, R is the gas constant, T is the temperature and Δn the amount of generated oxygen. In a series of concentration-dependent measurements, the highest TON obtained at one single concentration was reported. TOFs were determined for each measurement. In series with varying solvent ratios, TOFs for single experiments at various solvent ratios were calculated from the obtained initial rates by linear regression through the first linear part of the oxygen evolution curve at the very beginning of catalysis. In series of multiple measurements of different concentration at a fixed solvent ratio, the reported TOF is the slope of the linear regression of the plot of the initial rates vs the amount of catalyst.

Photocatalytic water oxidation

Photocatalytic water oxidation experiments were carried out in a transparent and temperature-controlled reaction chamber at 20 °C. The chamber was equipped with a Clark electrode (Oxygraph Plus Clark-electrode system from Hansatech) for oxygen detection in solution. Irradiation of the samples was performed by a 150 W xenon lamp (Newport) with a 400 nm cutoff filter calibrated to an intensity of 100 mW cm^{-1}. Light intensity calibration inside the reaction chamber was carried out using a PM 200 optical power meter with a S121C sensor (Thorlabs) combined with a CCS 200/M wide range spectrometer (Thorabs). A stock solution of the photosensitizer and sodium persulfate in a 1:1 acetonitrile/water mixture (pH 7, phosphate buffer) was prepared in the dark directly before starting a series of experiments. An aliquant of the stock solution (1.5 mL) was mixed with a varying amount of catalyst concentration (0.5 mL) inside the reaction chamber in the dark for 45 seconds prior to illumination. For all experiments, the amount of photosensitizer (c = 1.5 mM) and sodium persulfate (c = 37 mM) was kept constant and a blank without any catalyst was subtracted before evaluating the data. The TON was determined by evaluating the highest amount of oxygen in solution during each experiment and dividing by the amount of catalyst used. The reported TON value corresponds to the highest TON at one concentration. For the determination of TOF, oxygen evolution was plotted versus the reaction time of the experiment. The oxygen evolution after a short induction period (~1 s) was fitted by linear regression analysis during the first five to ten seconds of catalysis to give the initial rates of catalysis. The slope of a plot of the initial rates versus the catalyst concentration gives the TOF.

Synthesis of ligands and macrocyclic WOCs

New ligands **L2** and **L4** were synthesized according to Scheme S1. The reaction of commercially available 3-bromo-5-hydroxypyridine **2** and 1,3-dibromo-5-hydroxybenzene **3** with OEG-functionalized tosylate **4** according to literature procedures[7] afforded the respective intermediates **5** and **6**. Subsequent Suzuki-Miyaura cross-coupling reaction of the latter compounds with 1,3-benzenediboronic acid bis(pinacol) ester **7** and 4-pyridineboronic acid pinacol ester **8**, respectively, gave OEG-functionalized bidentate ligands **L2** and **L4**.

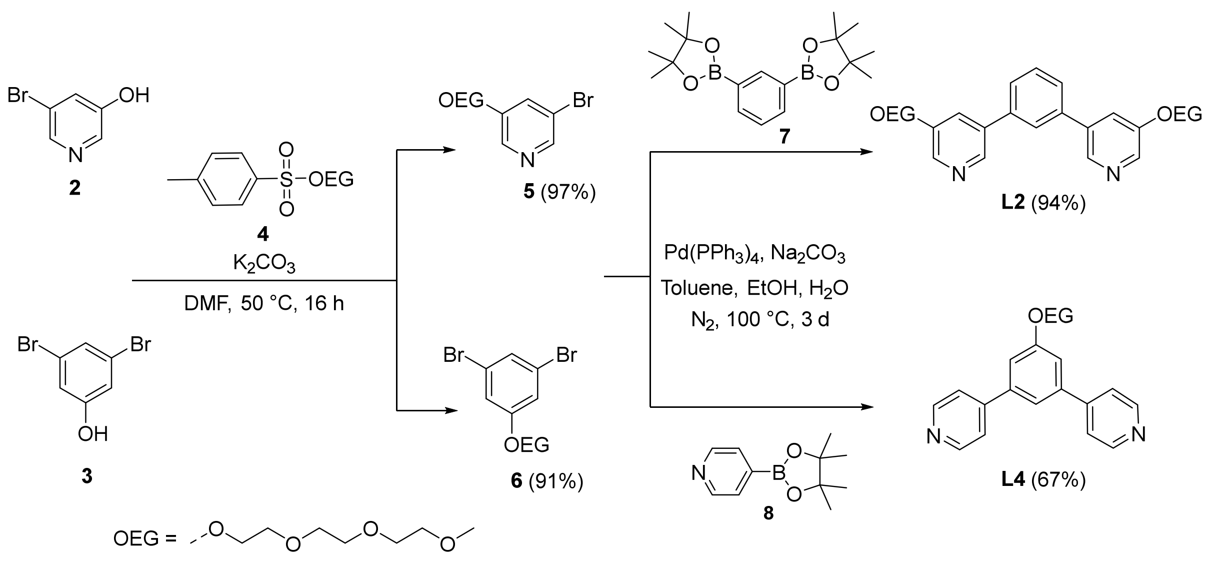

Scheme S1: Synthesis of axial ligands **L2** and **L4**. Compounds **2**, **3** and **8** were commercially available. Synthesis of **4**,[7a] **5**,[7b] **6**,[7c] and **7**,[7d] were performed according to literature.

1,3-Bis(5-(2-(2-(2-methoxyethoxy)ethoxy)ethoxy)pyridin-3-yl)benzene (L2)

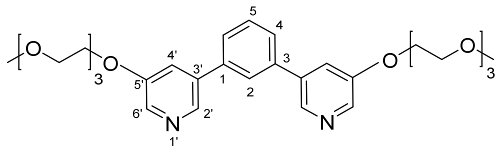

The reaction was carried out in an inert nitrogen gas atmosphere. A mixture of toluene (10.0 mL), ethanol (6.0 mL) and an aqueous sodium carbonate solution (2 M, 10.0 mL) was degassed by "pump-freeze-thaw" cycles. Subsequently, 1,4-bis(4,4,5,5-tetramethyl-1,3,2-dioxaborolan-2-yl)benzene (515 mg, 1.56 mmol, 1 equiv.), 3-bromo-5-(2-(2-(2-methoxy-ethoxy)ethoxy)ethoxy)pyridine (1.50 g, 4.68 mmol, 3 equiv.) and $Pd(PPh_3)_4$ (180 mg, 156 mol, 0.10 equiv.) were added. The reaction mixture was stirred for 3 d at 100 °C and the solvent was removed *in vacuo*. The product was purified by flash-column chromatography (SiO_2, EA 100% to EA:MeOH 8:2). The desired compound **L2** was obtained as an off-white solid (813 mg, 1.46 mmol, 94%).

Melting point: 57-58 °C. ^{1}H NMR (400 MHz, CD_2Cl_2): δ = 8.50 (d, $^4J_{H\text{-}H}$ = 1.44 Hz, 2H, Ar-$H_{2'}$), 8.32 (d, $^4J_{H\text{-}H}$ = 2.74 Hz, 2H, Ar-$H_{6'}$), 7.82 (dt, $^4J_{H\text{-}H}$ = 1.82 Hz, $^5J_{H\text{-}H}$ = 0.50 Hz, 1H, Ar-H_5), 7.66-7.57 (m, 3H, Ar-$H_{2,4'}$), 7.50 (dd, 2H, $^4J_{H\text{-}H}$ = 2.75 Hz, $^4J_{H\text{-}H}$ = 0.50 Hz, Ar-H_4), 4.27-4.25 (m, 4H, Ar-O-CH_2-CH_2-O), 3.88-3.86 (m, 4H, Ar-O-CH_2-CH_2O), 3.70-3.67 (m, 4H, Ar-(O-C_2H_4)-O-CH_2-R), 3.64-3.61 (m, 4H, Ar-(O-C_2H_4)-O-CH_2-CH_2), 3.59-3.57 (m, 4H, Ar-(O-C_2H_4)$_2$-O-CH_2-R), 3.50-3.47 (m, 4H Ar-(O-C_2H_4)$_2$-O-CH_2-CH_2-R), 3.32 (s, 6H, OCH_3) ppm. ^{13}C NMR (100 MHz, CD_2Cl_2): δ = 155.7, 141.3, 139.1, 137.5, 137.4, 130.3, 127.6, 127.7, 120.2, 72.4, 71.4, 71.0,

71.0, 70.1, 68.6, 59.2 ppm. HR-MS (ESI$^+$, MeCN/$CHCl_3$ 1:1): *m/z* calculated for $[M+H]^+$ ($[C_{30}H_{41}N_2O_8]^+$): 557.2857, found: 557.2877.

4,4'-(5-(2-(2-(2-Methoxyethoxy)ethoxy)ethoxy)-1,3- phenylene)dipyridine (L4)

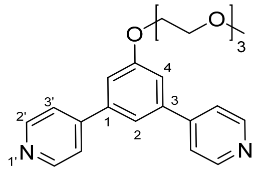

The reaction was carried out in an inert nitrogen gas atmosphere. A mixture of toluene (16.0 mL), ethanol (2.0 mL) and an aqueous sodium carbonate solution (2 M, 16.0 mL) was degassed by "pump-freeze-thaw" cycles. Subsequently, 1,3-dibromo-5-(2-(2-(2-methoxy-ethoxy)ethoxy)ethoxy)benzene (1.50 g, 3.77 mmol, 0.33 equiv.), 4 (2.34 g, 11.4 mmol, 1 equiv.) and $Pd(PPh_3)_4$ (1.32 g, 1.14 mmol, 0.1 equiv.) were added and the reaction mixture was stirred for 3 d at 100 °C. The solvent was removed *in vacuo* and the product was purified by flash-column chromatography (SiO_2, EA 100% to EA:MeOH 8:2). Compound **L4** was obtained as a pale yellow solid (996 mg, 2.53 mmol, 67%).

Melting point: 65-66 °C. ^{1}H NMR (400 MHz, CD_2Cl_2): δ = 8.67 (dd, $^4J_{H-H}$ = 1.47 Hz, $^3J_{H-H}$ = 4.72 Hz, 4H, Ar-$H_{2'}$), 7.57 (dd, $^4J_{H-H}$ = 1.58 Hz, $^3J_{H-H}$ = 4.73 Hz, 4H, Ar-$H_{3'}$), 7.51 (s, 1H, Ar-H_2), 7.28 (d, $^4J_{H-H}$ = 1.54 Hz, 2H, Ar-H_4), 4.28-4.26 (m, 2H, Ar-O-CH_2-CH$_2$O), 3.89-3.87 (m, 2H, Ar-O-CH$_2$-CH_2-(O-C$_2$H$_4$)$_2$), 3.72-3.69 (m, 2H, Ar-O-(CH$_2$)$_2$-(O-C$_2$$H_4$)$_2$), 3.64-3.62 (m, 2H, Ar-O-(CH$_2$)$_2$-(O-C$_2$$H_4$)$_2$), 3.60-3.58 (m, 2H, Ar-O-(CH$_2$)$_2$-(O-C$_2$$H_4$)$_2$), 3.50-3.48 (m, 2H, Ar-O-(CH$_2$)$_2$-(O-C$_2$$H_4$)$_2$), 3.32 (s, 3H, O-C$H_3$) ppm. ^{13}C NMR (100 MHz, CD_2Cl_2): δ = 160.4, 150.7, 148.0, 140.9, 122.1, 118.8, 114.2, 72.3, 71.2, 70.9, 70.8, 70.0, 68.4, 59.0 ppm. HR-MS (ESI$^+$, MeCN/$CHCl_3$ 1:1): *m/z* calculated for $[M+H]^+$ ($[C_{23}H_{27}N_2O_4]^+$): 395.1965, found: 395.1967.

Synthesis of Ru(bda) macrocycles

OEG-MC2

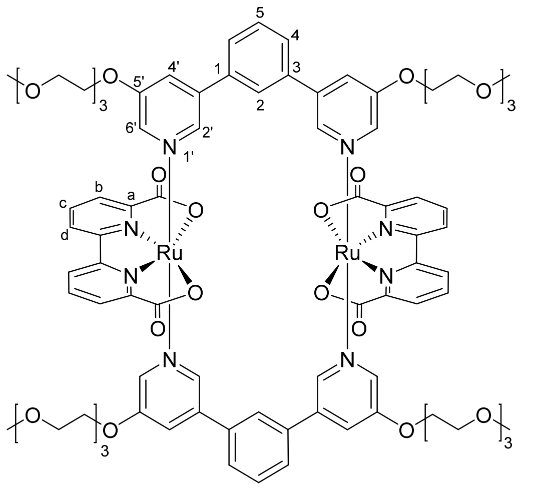

Ru(bda)(dmso)$_2$ (15.0 mg, 30.0 µmol, 1 equiv.) and ligand **L2** (16.7 mg, 30.0 µmol, 1 equiv.) were dissolved in degassed H_2O (12 mL) and stirred at 95 °C for 72 h in a nitrogen inert gas atmosphere. The solvent was removed by filtration and the remaining solid was redissolved in DCM/MeOH (5:3) and filtered over aluminum oxide (neutral, 15% w/w H_2O). Further purification by size exclusion chromatography (BioBeads S-X1, DCM/MeOH 9:1) and subsequent recycling GPC cycles ($CHCl_3$/MeOH 9:1) yielded in 7.0 mg (3.9 µmol, 26%) of macrocycle **OEG-MC2**.

Melting point: > 300 °C. ^{1}H NMR (400 MHz, CD_2Cl_2 / MeOD): δ = 9.41 (s, 2H, Ar-H_d), 8.12-8.11 (m, 6H, Ar-$H_{b,6',2'}$), 7.49-7.45 (m, 3H, Ar-$H_{c,2}$), 7.35 (s, 2H, Ar-$H_{4'}$), 7.21 (s, 2H, Ar-H_4), 7.12 (s, 1H, Ar-H_5), 4.44 (s, 4H, Ar-O-CH_2-CH$_2$-O), 4.06-4.03 (m, 4H, Ar-O-CH$_2$-CH_2-O), 3.73-3.71 (m, 4H, Ar-O-(CH$_2$)$_2$-(O-C$_2H_4$)$_2$), 3.58-3.52 (m, 8H, Ar-O-(CH$_2$)$_2$-(O-C$_2H_4$)$_2$), 3.46-3.44 (m, 4H, -(O-C$_2H_4$)-OCH$_3$), 3.28 (s, 6H, OCH_3) ppm. ^{13}C NMR (100 MHz, CD_2Cl_2 / MeOD): δ = 174.7, 160.9, 157.6, 156.6, 143.4, 140.5, 138.7, 137.8, 134.4, 134.1, 131.1, 129.3, 127.0, 125.3, 121.6, 72.4, 71.3, 71.1, 70.9, 69.9, 69.1, 59.1 ppm. HR-MS (ESI$^+$, MeCN/$CHCl_3$ 1:1): *m/z* calculated for $[M+Na]^+$ ($[C_{84}H_{92}N_8O_{24}Ru_2]^+$): 1823.4204, found: 1823.4219.

OEG-MC4

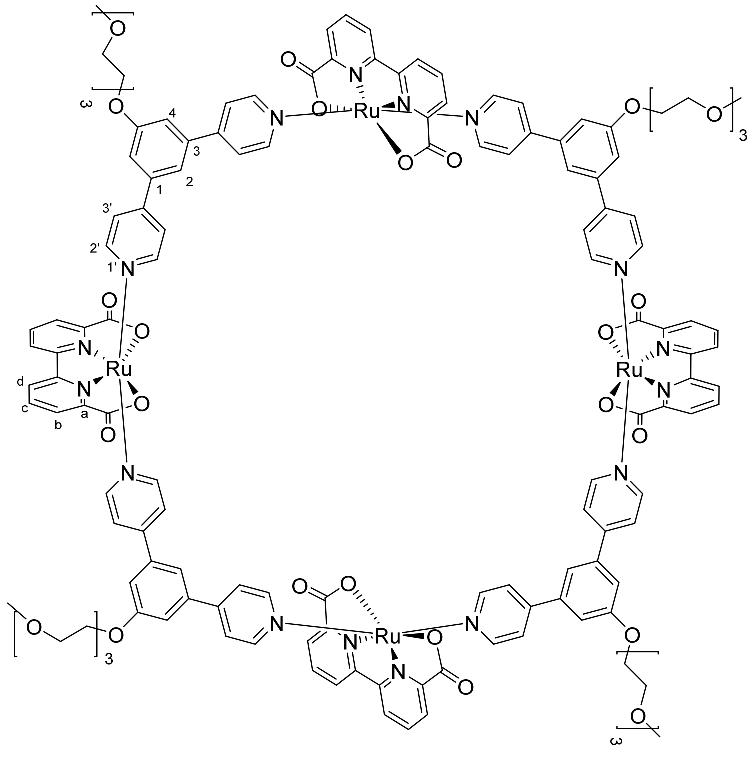

Ru(bda)(dmso)$_2$ (31.5 mg, 63.5 µmol, 1 equiv.) and ligand **L4** (25 mg, 63.5 µmol, 1 equiv.) were dissolved in degassed ethane-1,2-diol (15 mL) and stirred at 95 °C for 24 h in a nitrogen inert gas atmosphere. The solvent was removed by filtration and the remaining solid was redissolved in DCM/MeOH (5:3) and filtered over aluminum oxide (neutral, 15% w/w H_2O). Further purification by size exclusion chromatography (BioBeads S-X1, DCM/MeOH 9:1) and subsequent recycling GPC cycles ($CHCl_3$/MeOH 9:1) yielded in 7.5 mg (2.54 µmol, 16%) of **OEG-MC4**.

Melting point: > 300 °C. ^{1}H NMR (400 MHz, CD_2Cl_2 / MeOD / CF_3CD_2OD): δ = 8.46 (d, $^3J_{H-H}$ = 7.40 Hz, 8H, Ar-H_d), 7.98 (d, $^3J_{H-H}$ = 6.66 Hz, 8H, Ar-H_b), 7.83-7.78 (m, 24H, Ar-$H_{c,2'}$), 7.32 (d, $^3J_{H-H}$ = 6.66 Hz, 16H, Ar-$H_{3'}$), 7.23 (s, 4H, Ar-H_2), 7.10 (s, $^3J_{H-H}$ = 6.66 Hz, 8H, Ar-H_4), 4.11-4.07 (m, 8H, Ar-O-CH_2-CH$_2$-O), 3.76-3.72 (m, 8H, Ar-O-CH$_2$-CH_2-O), 3.60-3.65 (m, 8H, Ar-O-(CH$_2$)$_2$-(O-C$_2H_4$)$_2$), 3.54-3.48 (m, 16H, Ar-O-(CH$_2$)$_2$-(O-C$_2H_4$)$_2$), 3.41-3.39 (m, 8H, Ar-O-(CH$_2$)$_2$-(O-C$_2H_4$)$_2$), 3.22 (s, 12H, O-CH_3) ppm. ^{13}C NMR (100 MHz, CD_2Cl_2 / MeOD / CF_3CD_2OD): δ = 174.1, 160.7, 160.3, 157.3, 152.8, 148.5, 139.4, 132.3, 126.6, 125.1, 123.2, 118.7, 114.9, 72.3, 71.1, 70.9, 70.8, 70.0, 68.4, 58.9 ppm. HR-MS (ESI$^+$, MeCN/$CHCl_3$ 1:1): *m/z* calculated for [M]$^+$ ([$C_{140}H_{128}N_{16}O_{32}Ru_4$]$^+$): 2952.5055, found: 2950.4640.

NMR spectra

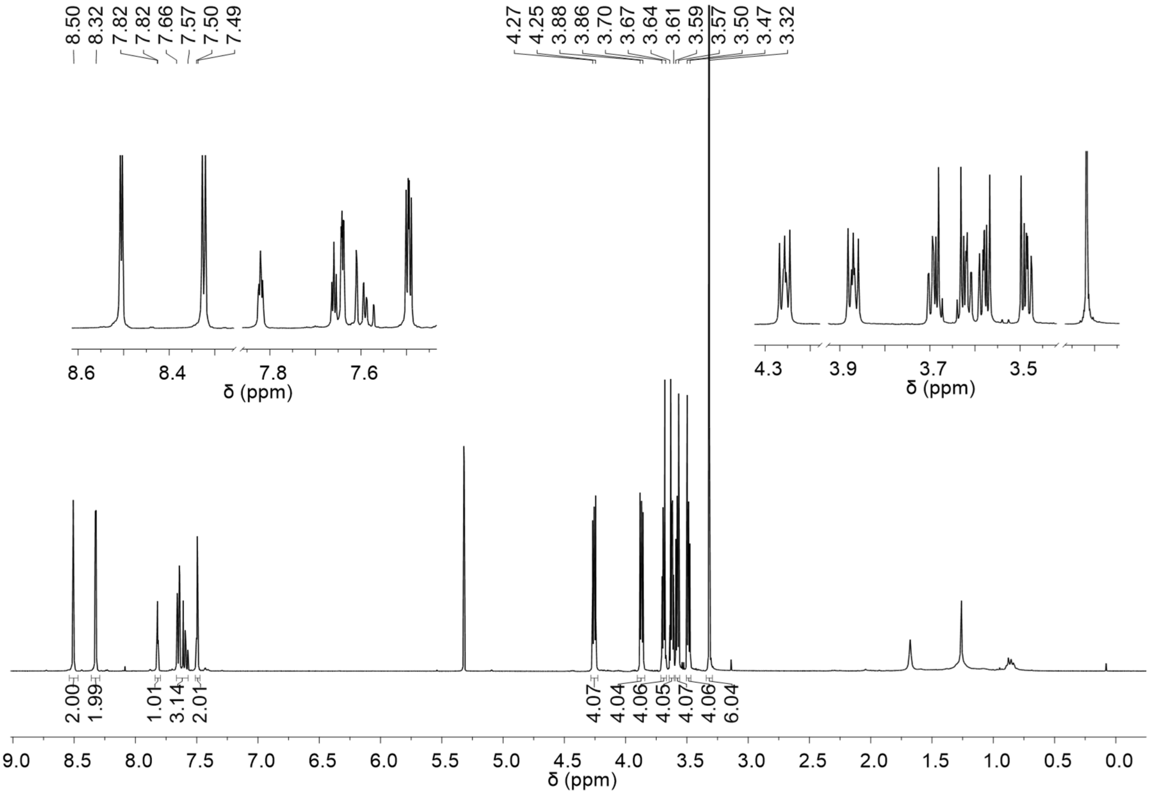

Figure S1: ^{1}H NMR spectrum (400 MHz, CD_2Cl_2) of **L2**.

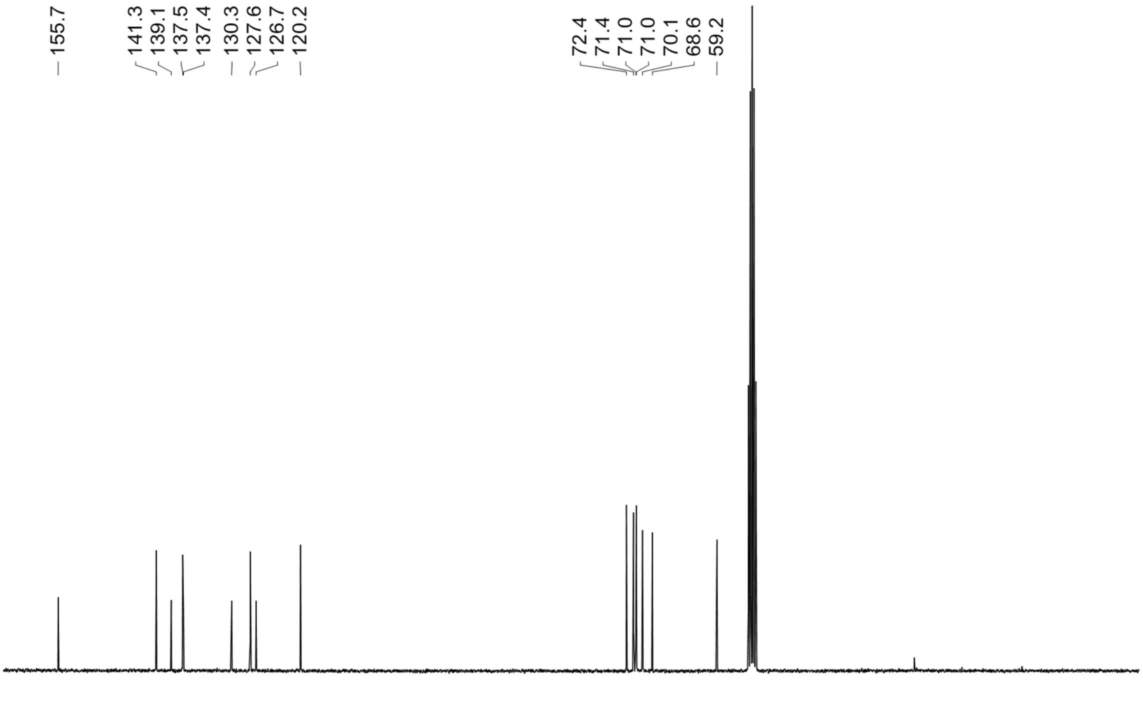

Figure S2: ^{13}C NMR spectrum (100 MHz, CD_2Cl_2) of **L2**.

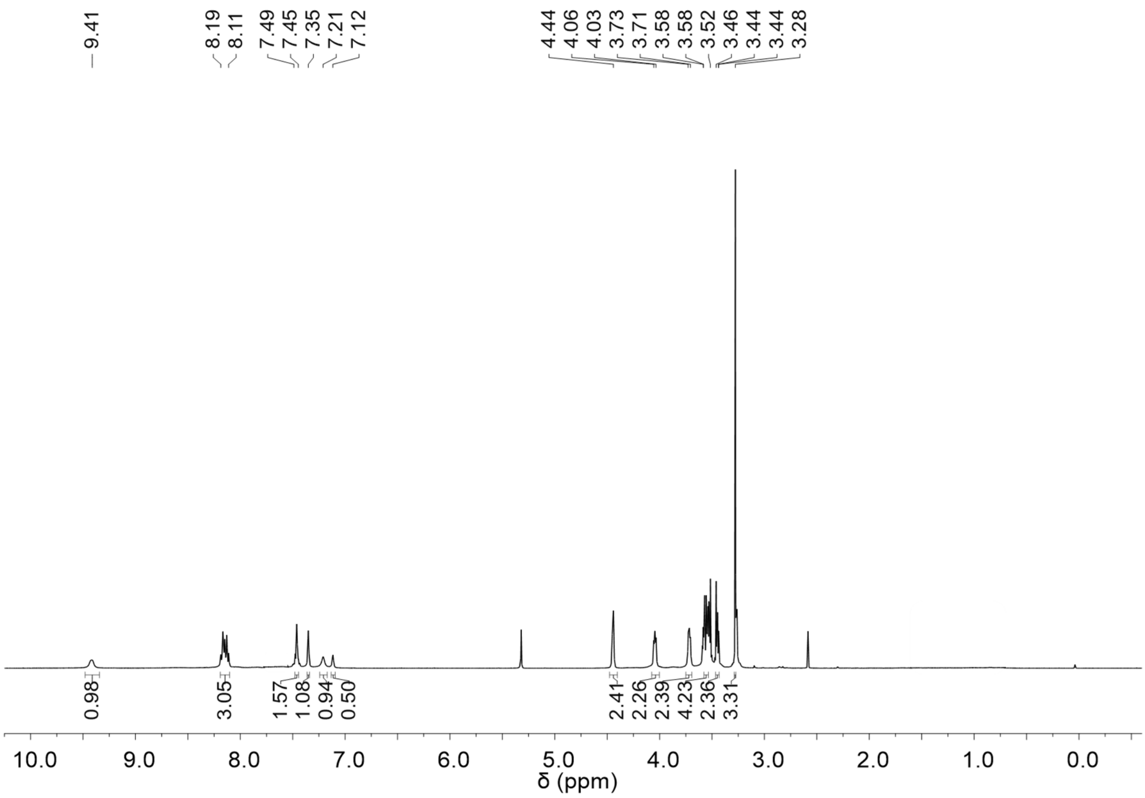

Figure S3: ^{1}H NMR spectrum (400 MHz, CD_2Cl_2/MeOD 5:3) of **OEG-MC2**. The integration of the signals is normalized for a monomeric unit.

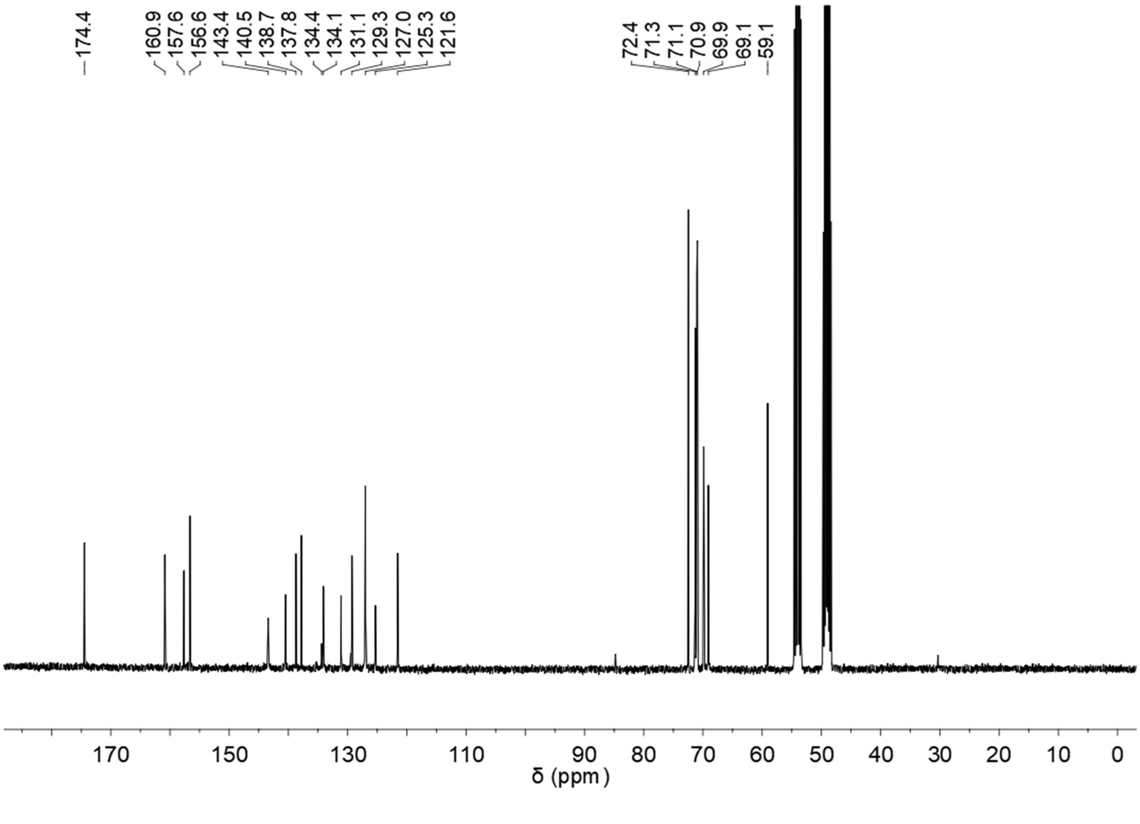

Figure S4: ^{13}C NMR spectrum (100 MHz, CD_2Cl_2/MeOD 5:3) of **OEG-MC2**.

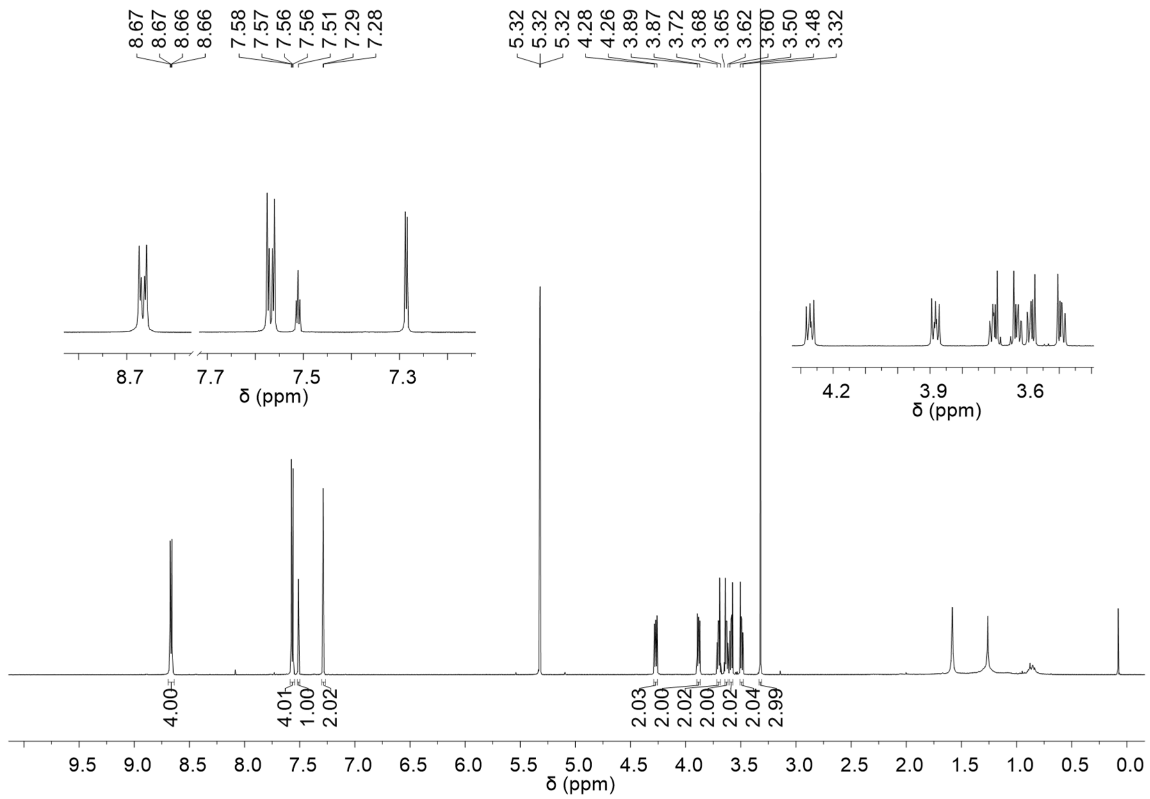

Figure S5: ^{1}H NMR spectrum (400 MHz, CD_2Cl_2) of **L4**.

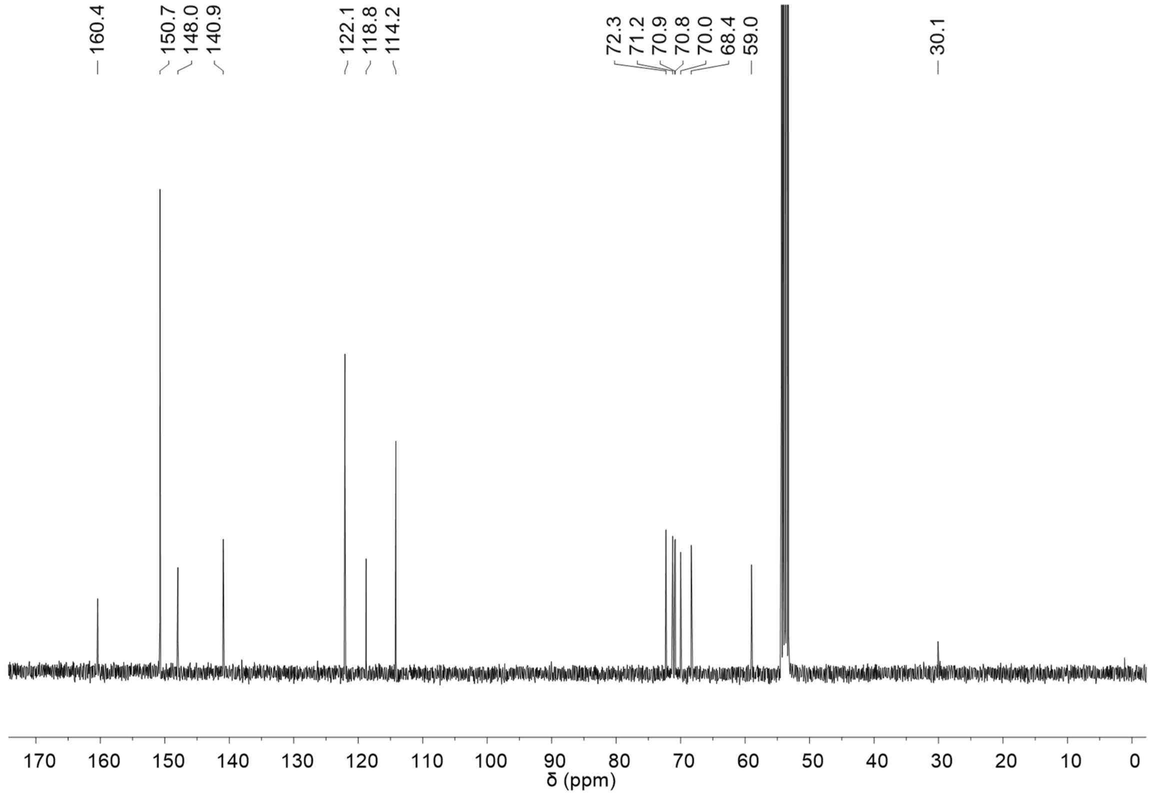

Figure S6: ^{13}C NMR spectrum (100 MHz, CD_2Cl_2) of **L4**.

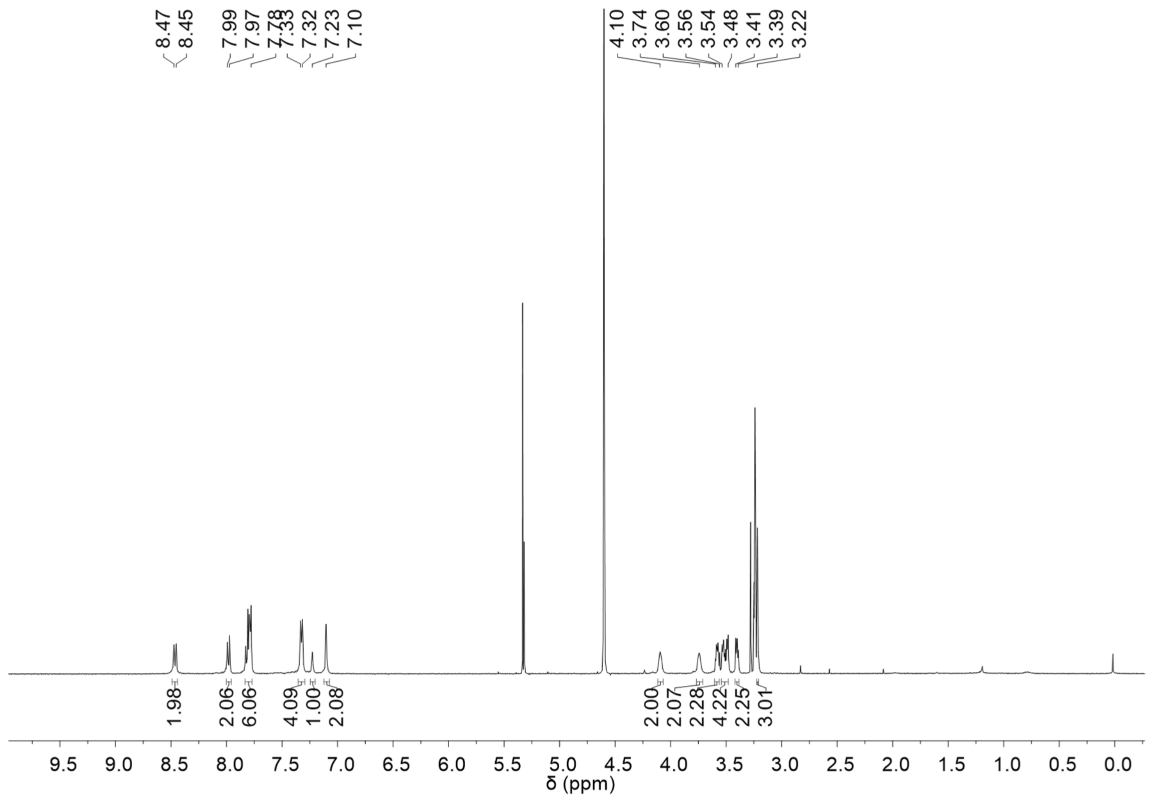

Figure S7: ^{1}H NMR spectrum (400 MHz, CD_2Cl_2/MeOD/TFE-d_3 5:3:2) of **OEG-MC4**. The integration of the signals is normalized for a monomeric unit.

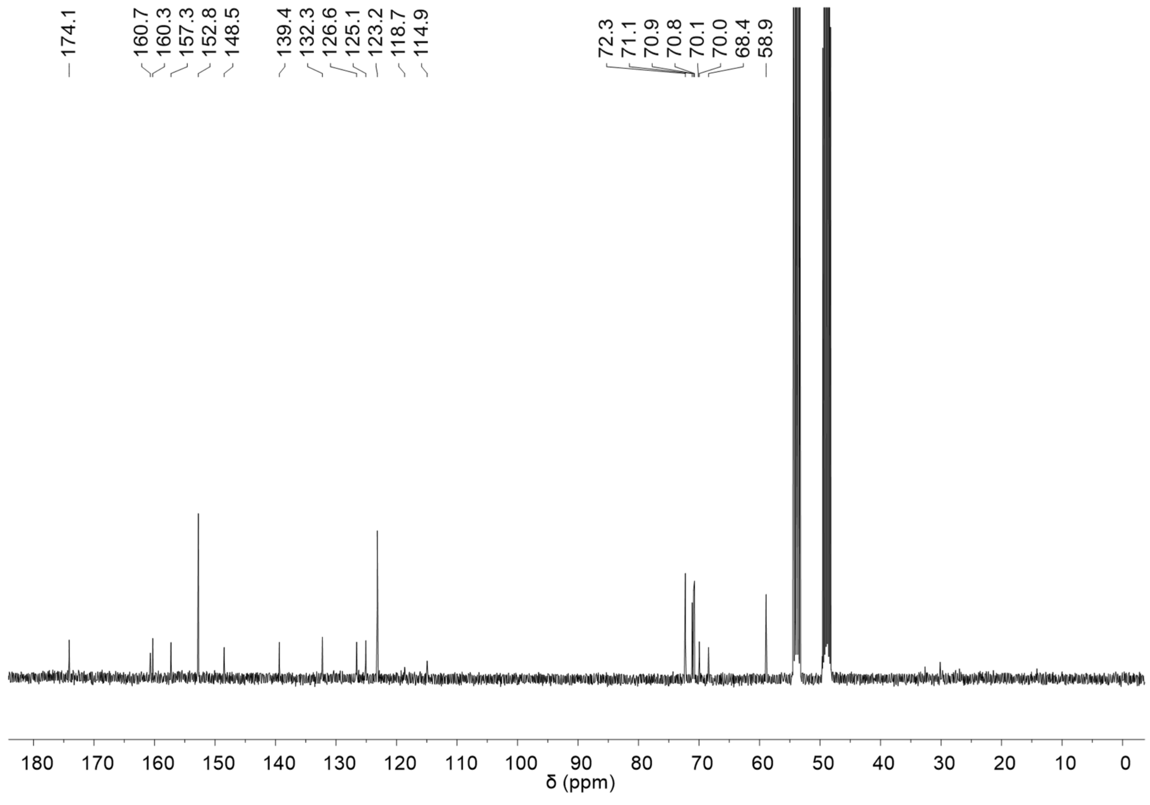

Figure S8: ^{13}C NMR spectrum (100 MHz, CD_2Cl_2/MeOD/TFE-d_3 5:3:2) of **OEG-MC4**.

HR mass spectra

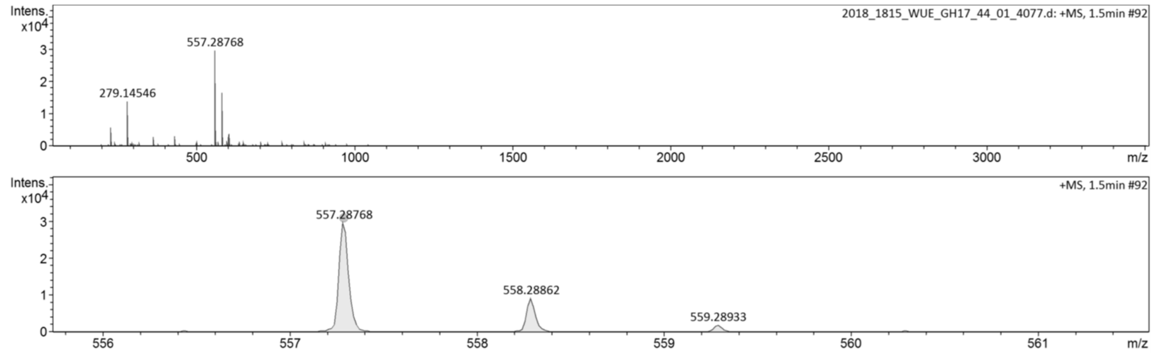

Figure S9: HR ESI mass spectrum (MeCN/$CHCl_3$ 1:1, positive mode) of **L2**.

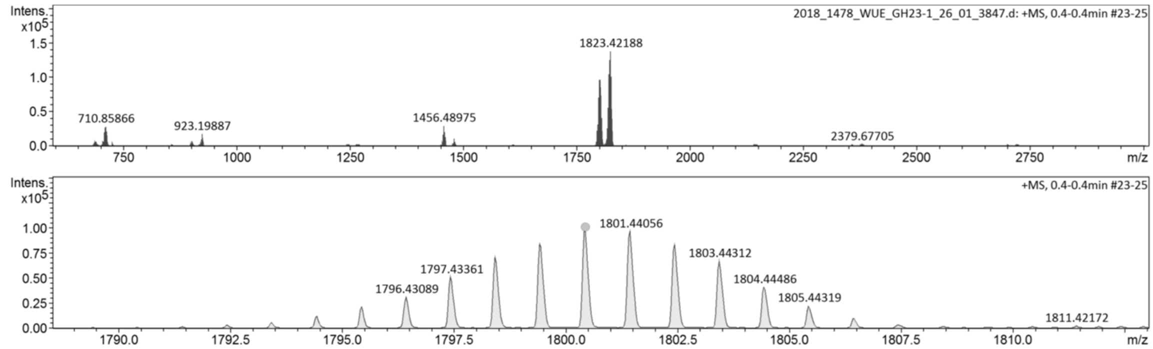

Figure S10: HR ESI mass spectrum (MeCN/$CHCl_3$ 1:1, positive mode) of **OEG-MC2**.

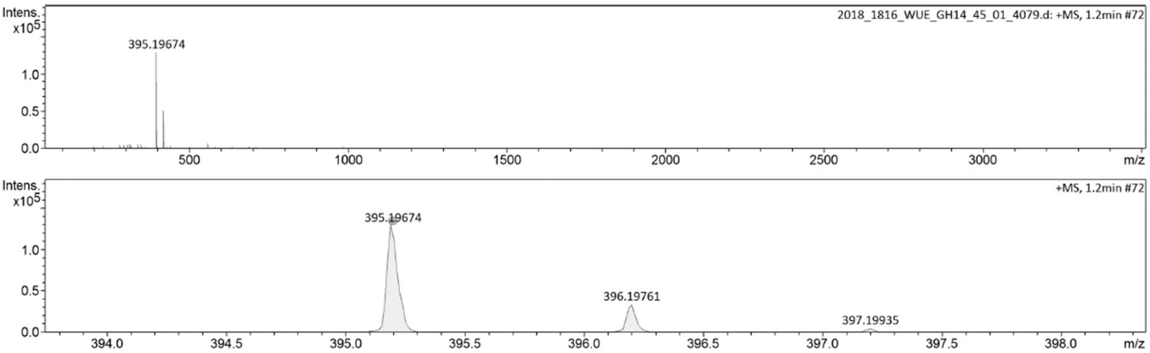

Figure S11: HR ESI mass spectrum (MeCN/$CHCl_3$ 1:1, positive mode) of **L4**.

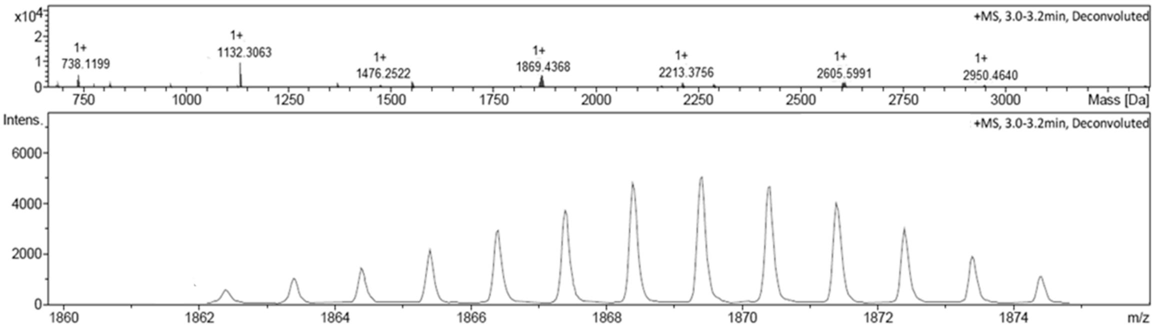

Figure S12: HR ESI mass spectrum (MeCN/$CHCl_3$ 1:1, positive mode) of **OEG-MC4**. Signal on the bottom belongs to [Ru(bda)$_2$(L4)$_3$] fragment. Calculated: *m*/*z* = 1869.8985, found: *m*/*z* = 1869.4368.

DOSY NMR spectra

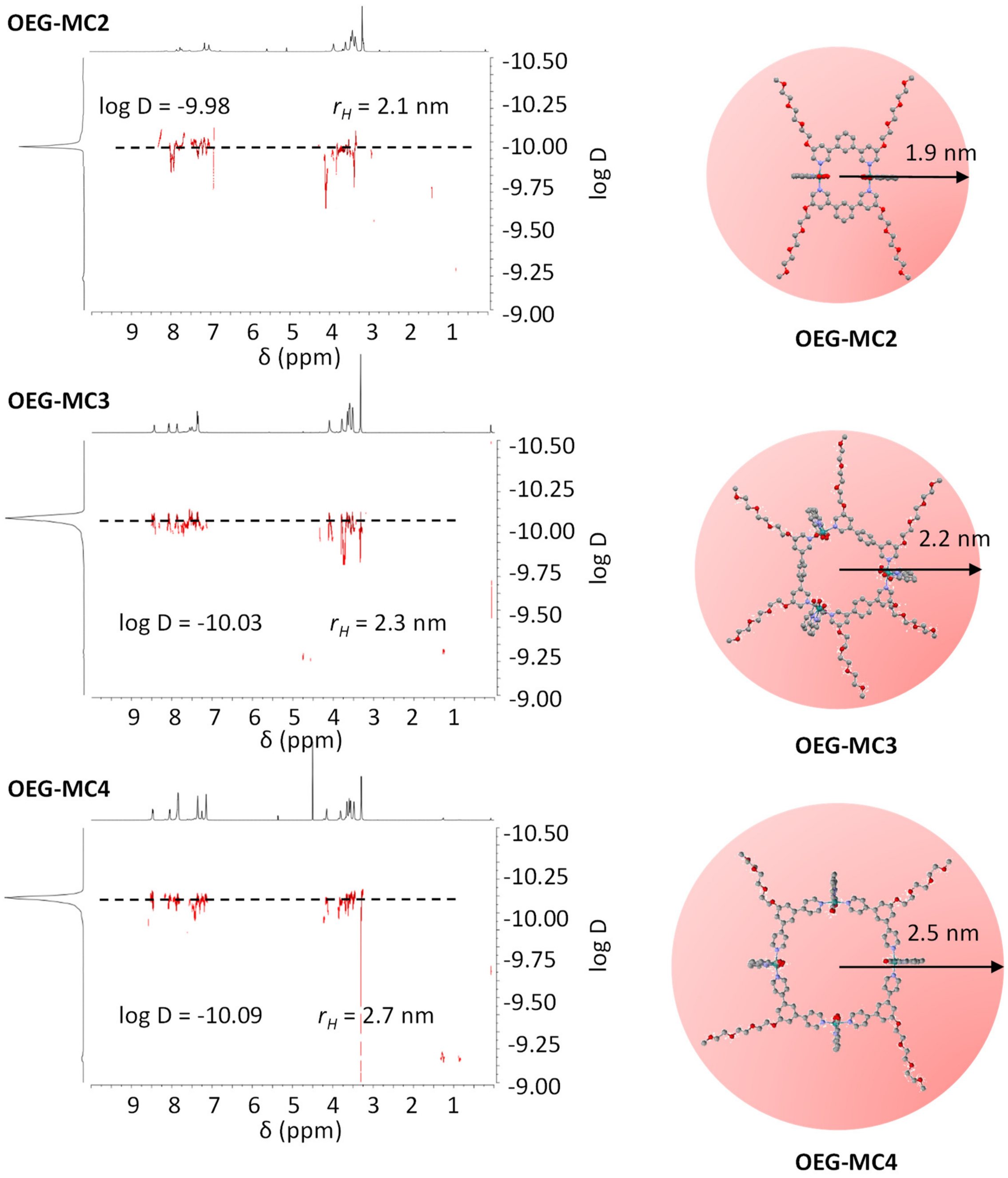

Figure S13: Left: DOSY NMR (400 MHz, rt) of **OEG-MC2, OEG-MC3** and **OEG-MC4** in CD_2Cl_2/MeOD/ TFE-d_3 (3:7:10). Right: Modelled structures and diameters of these compounds.

UV/vis absorption spectroscopy and spectroelectrochemical measurements

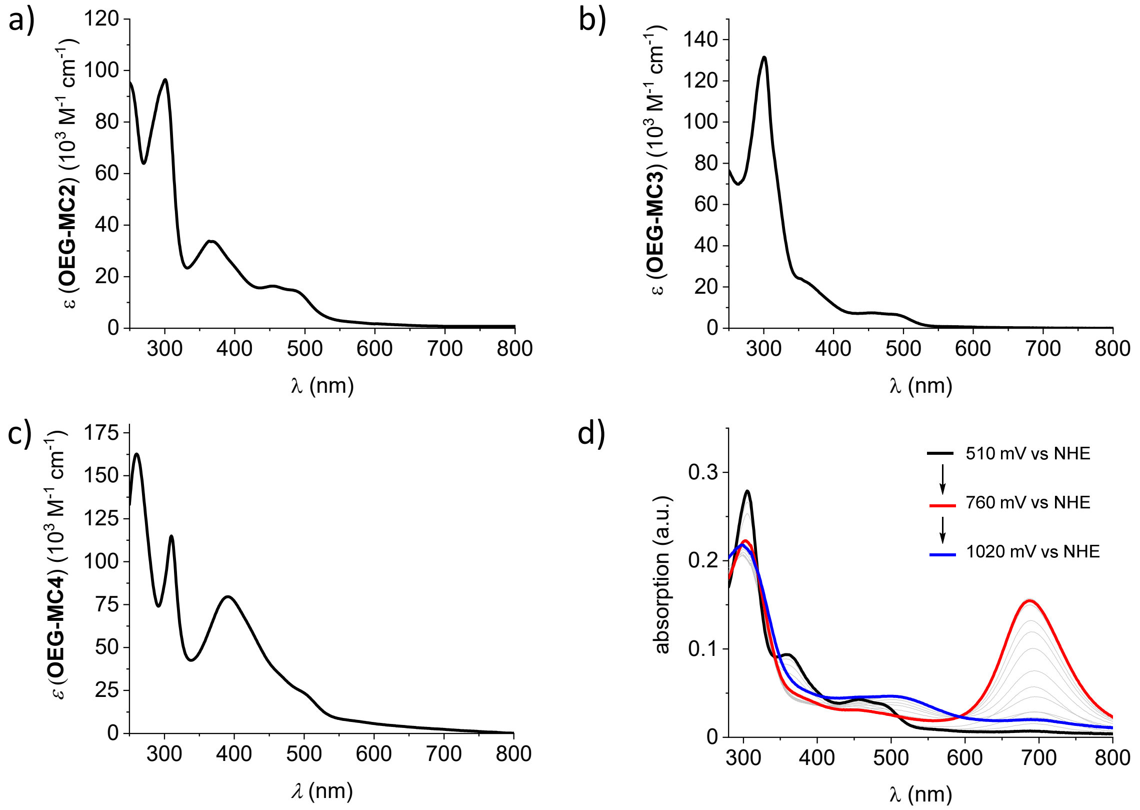

Figure S14: UV/Vis absorption spectra in MeCN/H_2O (phosphate buffer, pH 7, 0.1 M) (1:1) of a) **OEG-MC2** (c = 11), b) **OEG-MC3** (c = 10 µM), c) **OEG-MC4** (c = 8). d) Spectroelectrochemical studies of **OEG-MC3** (c = 219 µM) in a 1:1 mixture of TFE/H_2O (phosphate buffer, pH 7, 0.1 M).

Electrochemical studies

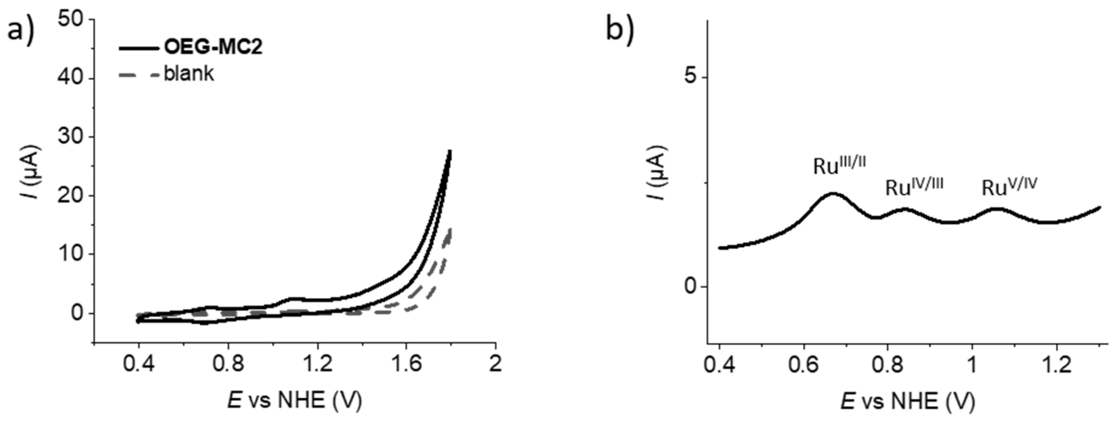

Figure S15: CV (scan rate: 100 mV/s) (a) and DPV (b) of **OEG-MC2** in TFE/H_2O 1:1 (pH 7, phosphate buffer), c = 0.25 mM.

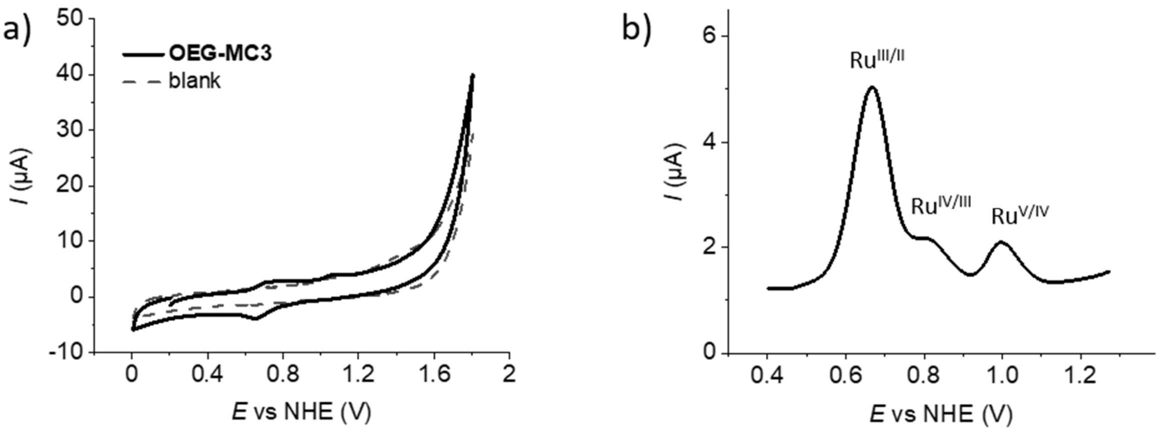

Figure S16: CV (scan rate: 100 mV/s) (a) and DPV (b) of **OEG-MC3** in TFE/H_2O 1:1 (pH 7, phosphate buffer), c = 0.25 mM.

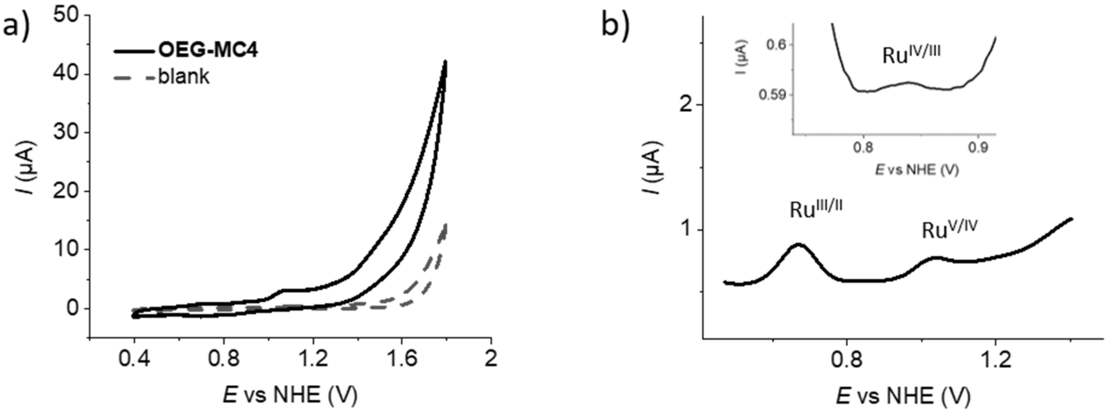

Figure S17: CV (scan rate: 100 mV/s) (a) and DPV (b) of **OEG-MC4** in TFE/H_2O 1:1 (pH 7, phosphate buffer), c = 0.25 mM.

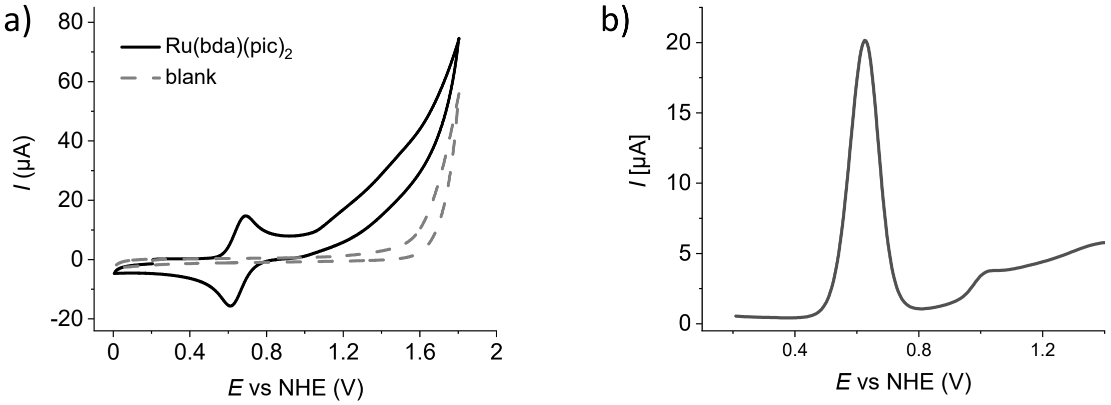

Figure S18: CV (scan rate: 100 mV/s) (a) and DPV (b) of Ru(bda)$(pic)_2$ in TFE/H_2O 1:1 (pH 7, phosphate buffer), c = 0.25 mM.

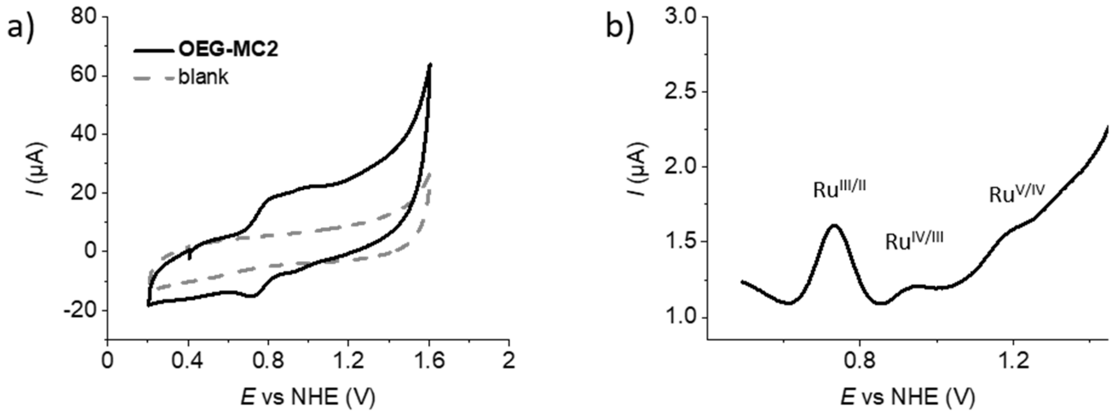

Figure S19: CV (scan rate: 100 mV/s) (a) and DPV (b) of **OEG-MC2** in TFE/H_2O 1:1 (pH 1, triflic acid), c = 0.25 mM.

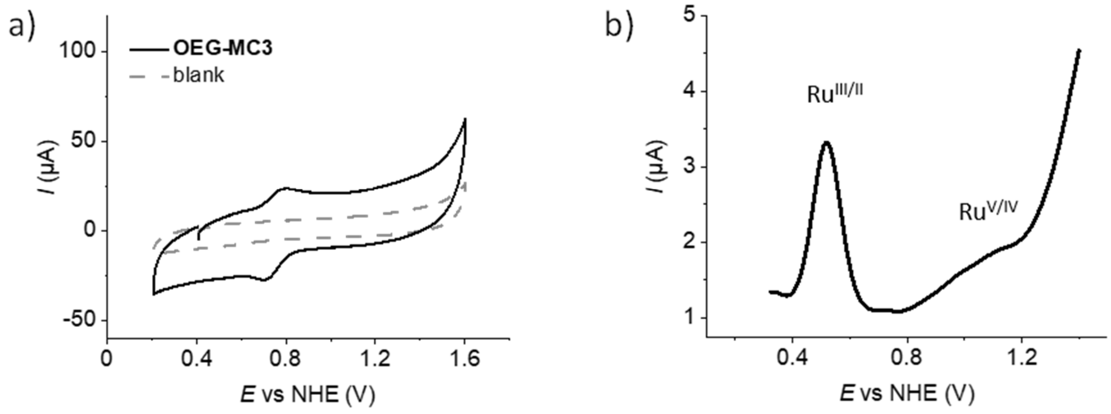

Figure S20: CV (scan rate: 100 mV/s) (a) and DPV (b) of **OEG-MC3** in TFE/H_2O 1:1 (pH 1, triflic acid), c = 0.25 mM. The Ru$^{IV/III}$ redox process gives only a very weak signal which connot be detected here.

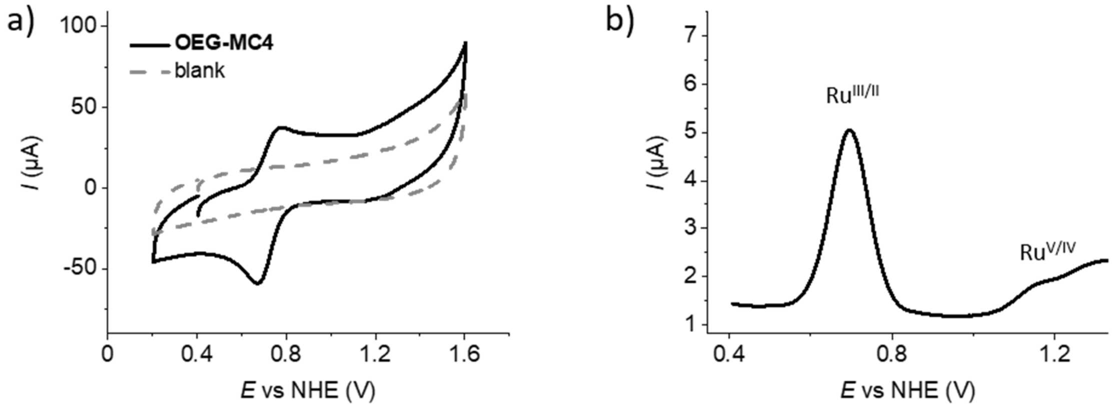

Figure S21: CV (scan rate: 100 mV/s) (a) and DPV (b) of **OEG-MC4** in TFE/H_2O 1:1 (pH 1, triflic acid), c = 0.25 mM. The Ru$^{IV/III}$ redox process gives only a very weak signal which connot be detected here.

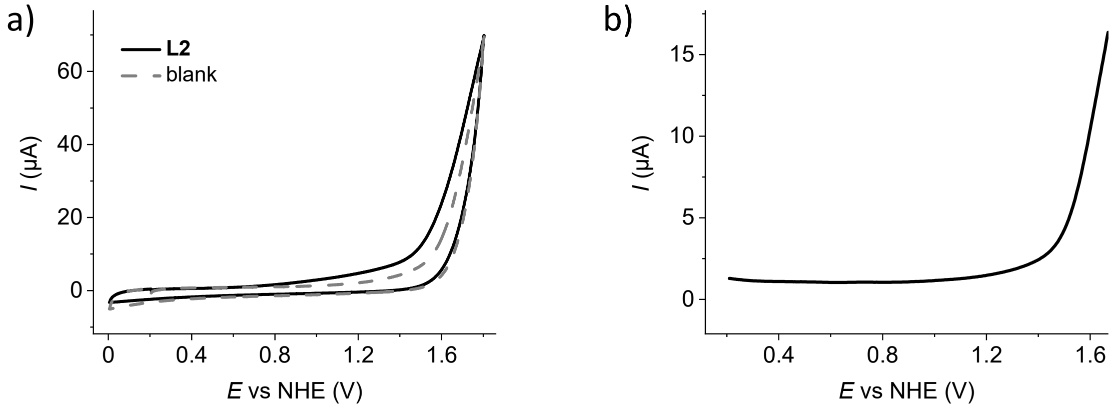

Figure S22: CV (scan rate: 100 mV/s) (a) and DPV (b) of **L2** in TFE/H_2O 1:1 (pH 7, phosphate buffer), c = 0.25 mM.

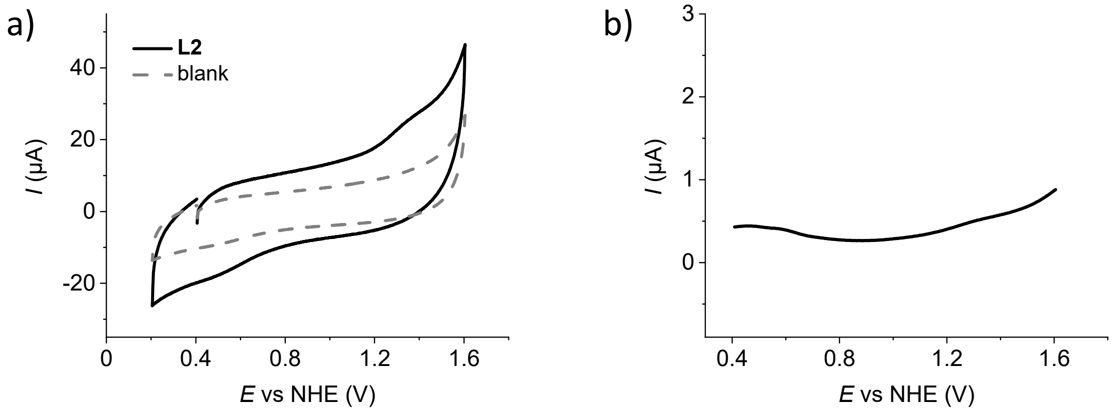

Figure S23: CV (scan rate: 100 mV/s) (a) and DPV (b) of **L2** in TFE/H_2O 1:1 (pH 1, triflic acid), c = 0.25 mM.

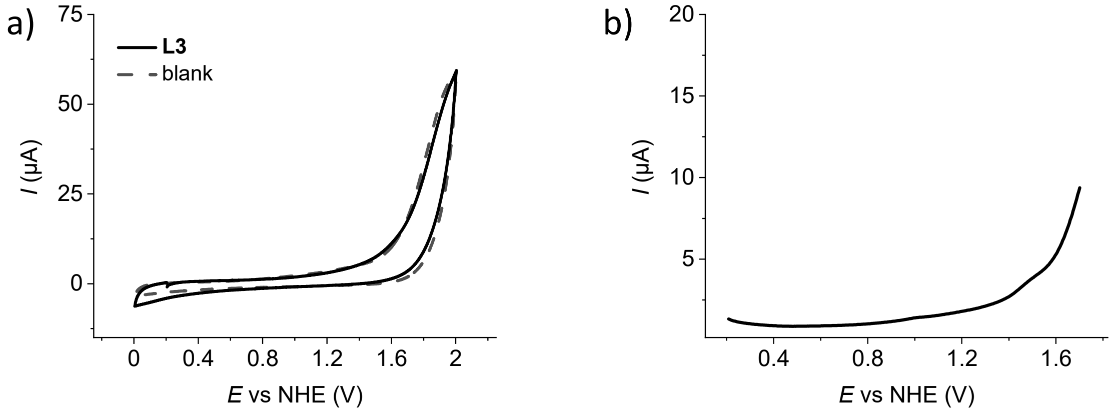

Figure S24: CV (scan rate: 100 mV/s) (a) and DPV (b) of **L3** in TFE/H_2O 1:1 (pH 7, phosphate buffer), c = 0.25 mM.

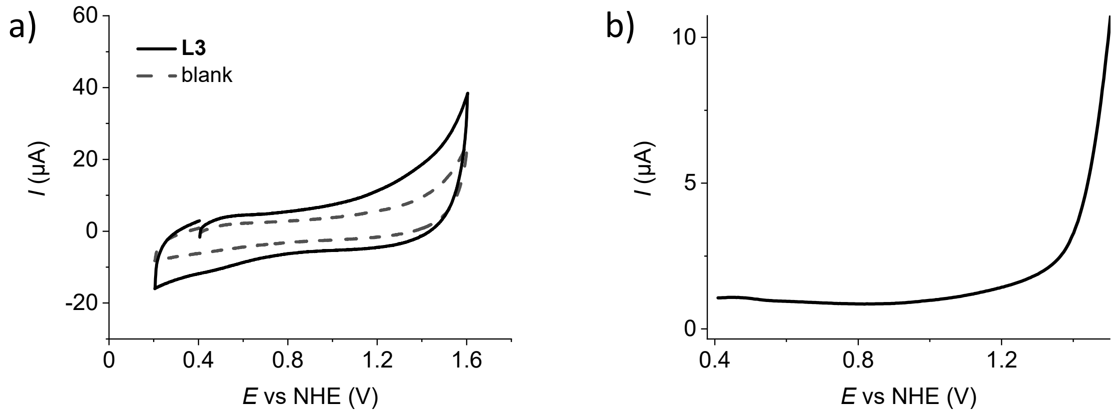

Figure S25: CV (scan rate: 100 mV/s) (a) and DPV (b) of **L3** in TFE/H_2O 1:1 (pH 1, triflic acid), *c* = 0.25 mM.

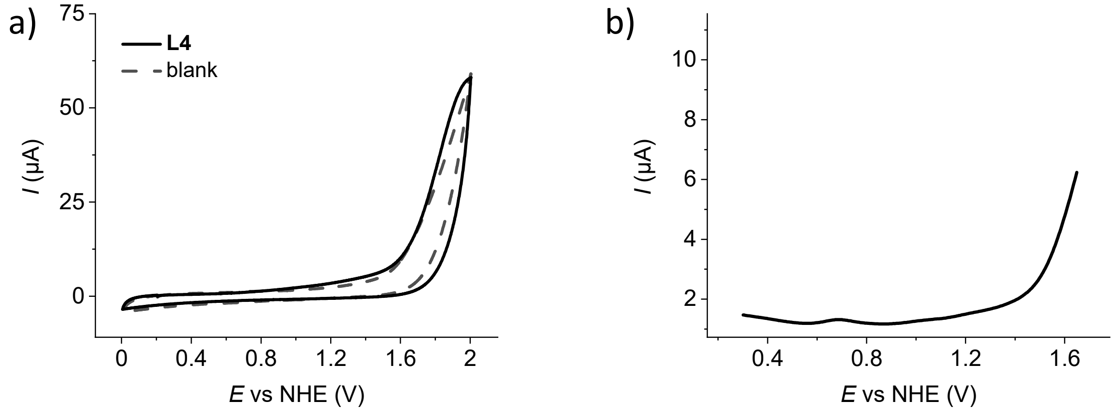

Figure S26: CV (scan rate: 100 mV/s) (a) and DPV (b) of **L4** in TFE/H_2O 1:1 (pH 7, phosphate buffer), *c* = 0.25 mM.

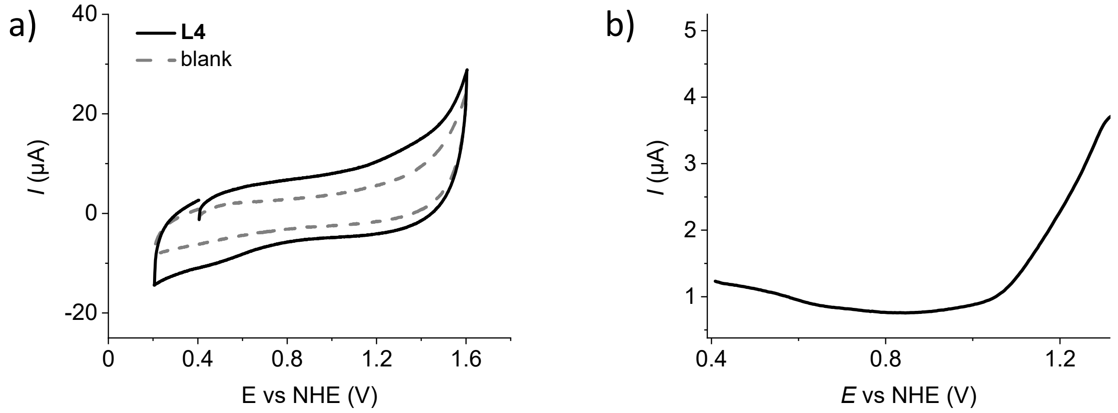

Figure S27: CV (scan rate: 100 mV/s) (a) and DPV (b) of **L4** in TFE/H_2O 1:1 (pH 1, triflic acid), *c* = 0.25 mM.

Chemical water oxidation

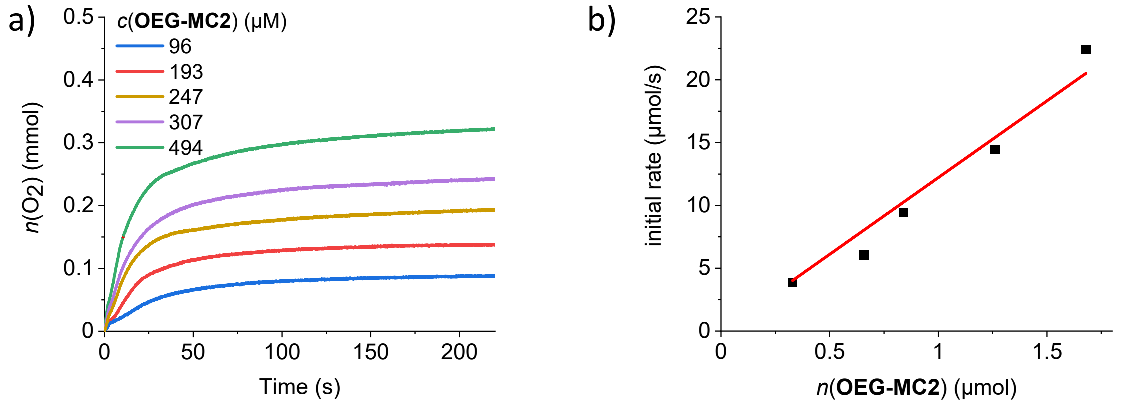

Figure S28: a) Concentration-dependent oxygen evolution curves for **OEG-MC2** in MeCN/H_2O 1:1 (pH 1, triflic acid). b) Plot of initial rates vs. catalyst amount with linear regression for the determination of TOF.

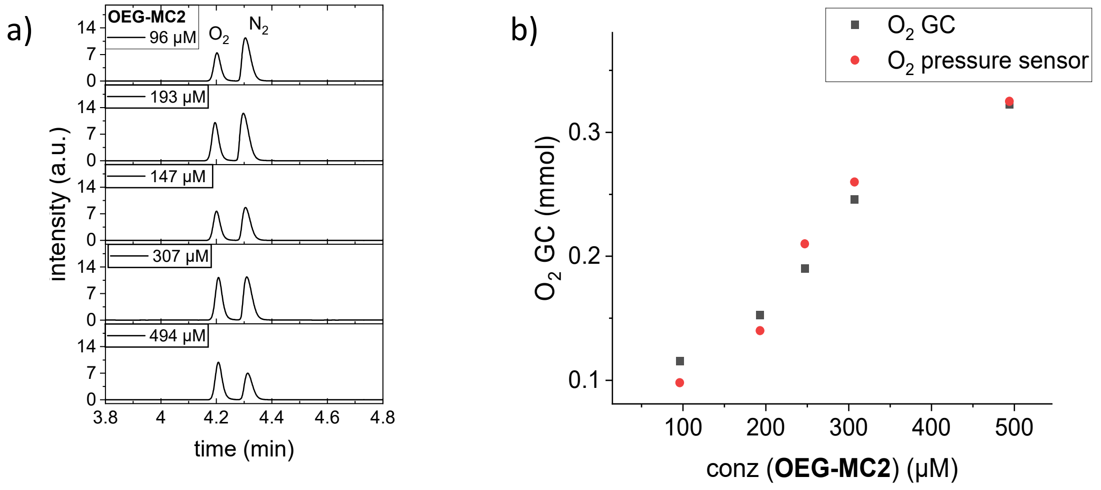

Figure S29: a) Chromatogram of headspace at the end of water oxidation experiments with **OEG-MC2** as WOC. b) Comparison of the amount of evolved oxygen determined by GC and with pressure sensor.

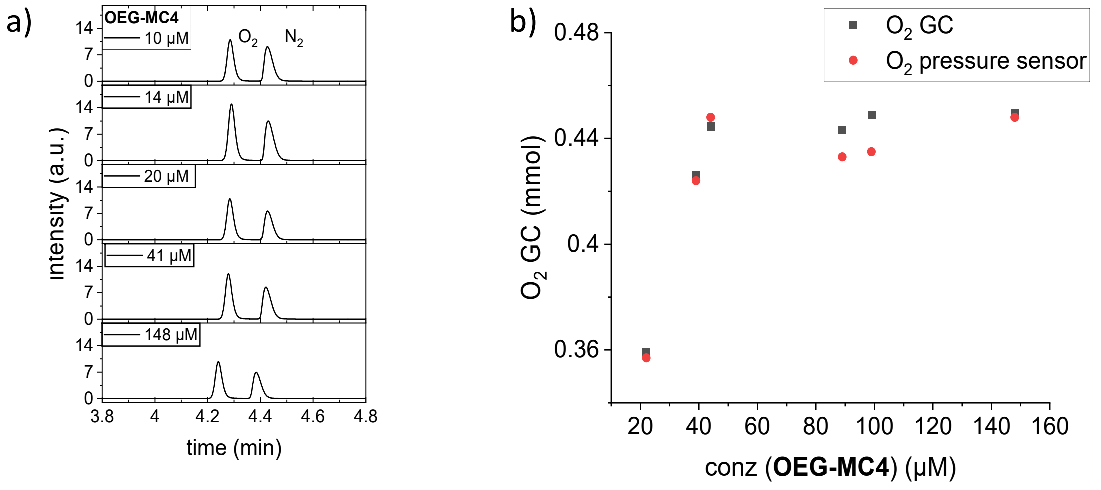

Figure S30: a) Chromatogram of headspace at the end of water oxidation experiments with **OEG-MC4** as WOC. b) Comparison of the amount of evolved oxygen determined by GC and with pressure sensor.

Photocatalytic water oxidation

a) b)

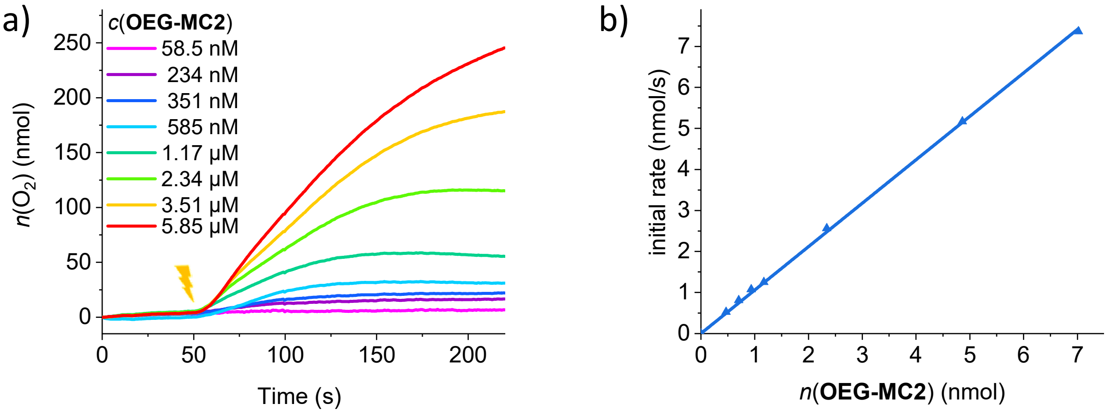

Figure S31: Concentration-dependent oxygen evolution curves for **OEG-MC2** in MeCN/H_2O 1:1 (phosphate buffer, pH 7). b) Plot of initial rates vs. catalyst amount with linear regression for the determination of TOF.

a) b)

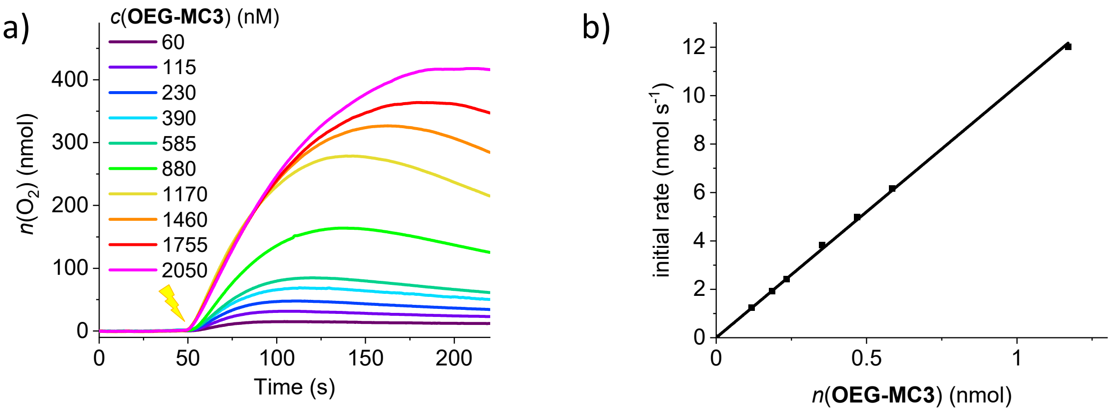

Figure S32: Concentration-dependent oxygen evolution curves for **OEG-MC3** in MeCN/H_2O 1:1 (phosphate buffer, pH 7). b) Plot of initial rates vs. catalyst amount with linear regression for the determination of TOF.

a) b)

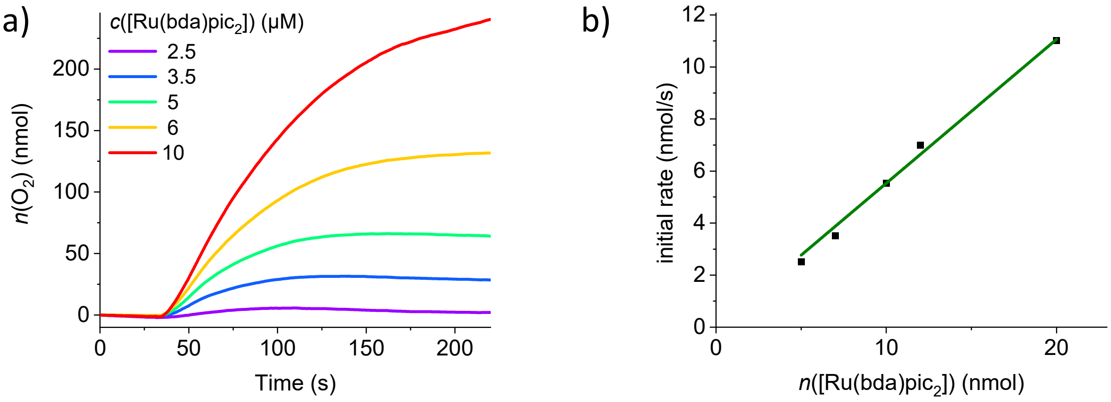

Figure S33: Concentration-dependent oxygen evolution curves for Ru(bda)(pic)$_2$ in MeCN/H_2O 1:1 (phosphate buffer, pH 7). b) Plot of initial rates vs. catalyst amount with linear regression for the determination of TOF. TON = 13. TOF = 0.6 s^{-1}.

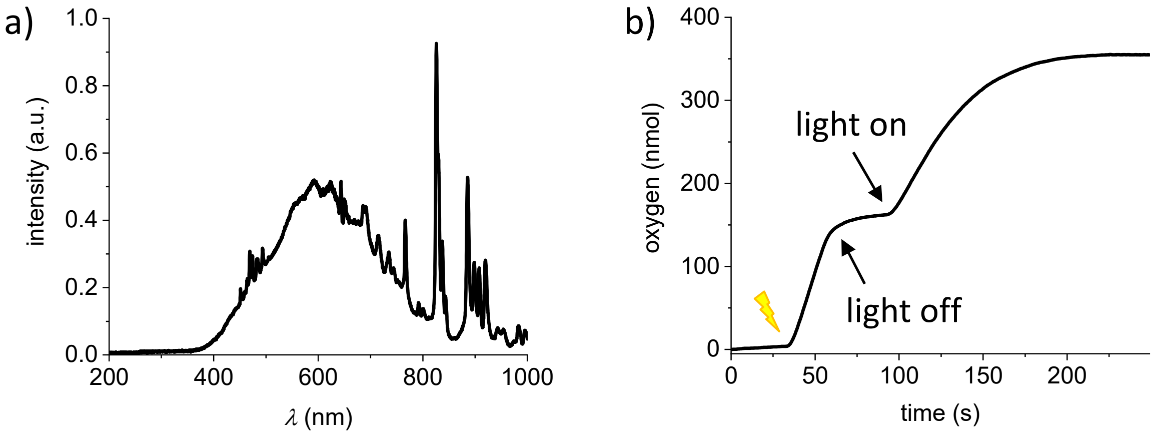

Figure S34: a) Emission profile of xenon lamp used for photocatalytic water oxidation experiments. b) Oxygen evolution curve vs time in photocatalytic water oxidation with **OEG-MC4** (*c* = 2000 nM) using Ru(bpy)$_3$ (*c* = 1.5 mM) as a photosensitizer and $Na_2S_2O_4$ (*c* = 37 mM) as a sacrificial electron acceptor. The lighting symbol indicates the time at which the sample was exposed to light for the first time after stirring for 30 seconds in the dark. The light was turned off after 30 seconds of illumination and no further oxygen was produced. After 30 seconds in the dark, the light was again turned on and the catalytic process was re-initiated, indicated by the rise in oxygen detected by the Clark electrode.

References

[1] a) C. L. Donnici, D. H. Máximo Filho, L. L. C. Moreira, G. T. d. Reis, E. S. Cordeiro, I. M. F. d. Oliveira, S. Carvalho, E. B. Paniago, *J. Braz. Chem. Soc.* **1998**, *9*, 455-460, b) T. M. Cassol, F. W. J. Demnitz, M. Navarro, E. A. d. Neves, *Tetrahedron Lett.* **2000**, *41*, 8203-8206.

[2] a) I. P. Evans, A. Spencer, G. Wilkinson, *J. Chem. Soc., Dalton Trans.* **1973**, 204-209;) E. Dulière, M. Devillers, J. Marchand-Brynaert, *Organometallics* **2003**, *22*, 804-811.

[3] a) F. Li, B. Zhang, X. Li, Y. Jiang, L. Chen, Y. Li, L. Sun, *Angew. Chem. Int. Ed.* **2011**, 12276-12279; *Angew. Chem.* **2011**, *123*, 12484-12487 b) Y. Gao, X. Ding, J. Liu, L. Wang, Z. Lu, L. Li, L. Sun, *J. Am. Chem. Soc.* **2013**, *135*, 4219-4222.

[4] L. Duan, A. Fischer, Y. Xu, L. Sun, *J. Am. Chem. Soc.* **2009**, *131*, 10397-10399.

[5] V. Kunz, M. Schulze, D. Schmidt, F. Würthner, *ACS Energy Lett.* **2017**, *2*, 288-293.

[6] M. Schulze, V. Kunz, P. D. Frischmann, F. Würthner, *Nat. Chem.* **2016**, *8*, 576-583.

[7] a) Z. Liu, B. X. Dong, M. Misra, Y. Sun, J. Strzalka, S. N. Patel, F. A. Escobedo, P. F. Nealey, C. K. Ober, *Adv. Funct. Mater.* **2019**, *29*, 1805220; b) W. Sun, Y. Wang, L. Ma, L. Zheng, W. Fang, X. Chen, H. Jiang, *J. Org. Chem.* **2018**, *83*, 14667-14675, c) S. Chen, S. Zhang, C. Bao, C. Wang, Q. Lin, L. Zhu, *Chem. Commun.* **2016**, *52*, 13132-13135; d) T. C. Lovell, C. E. Colwell, Lev N. Zakharov, R. Jasti, *Chem. Sci.* **2019**, *10*, 3786-3790.
